# RPA and Rad51 constitute a cell intrinsic mechanism to protect the cytosol from self DNA

**DOI:** 10.1038/ncomms11752

**Published:** 2016-05-27

**Authors:** Christine Wolf, Alexander Rapp, Nicole Berndt, Wolfgang Staroske, Max Schuster, Manuela Dobrick-Mattheuer, Stefanie Kretschmer, Nadja König, Thomas Kurth, Dagmar Wieczorek, Karin Kast, M. Cristina Cardoso, Claudia Günther, Min Ae Lee-Kirsch

**Affiliations:** 1 Department of Pediatrics, Medizinische Fakultät Carl Gustav Carus, Technische Universität Dresden, 01307 Dresden, Germany; 2 Department of Biology, Technische Universität Darmstadt, 64287 Darmstadt, Germany; 3 Department of Dermatology, Medizinische Fakultät Carl Gustav Carus, Technische Universität Dresden, 01307 Dresden, Germany; 4 Biotechnology Center, Technische Universität Dresden, 01307 Dresden, Germany; 5 Center for Regenerative Therapies, Technische Universität Dresden, 01307 Dresden, Germany; 6 Institute of Human Genetics, Heinrich-Heine-University, Medical Faculty, 40225 Düsseldorf, Germany; 7 Department of Gynecology, Medizinische Fakultät Carl Gustav Carus, Technische Universität Dresden, 01307 Dresden, Germany

## Abstract

Immune recognition of cytosolic DNA represents a central antiviral defence mechanism. Within the host, short single-stranded DNA (ssDNA) continuously arises during the repair of DNA damage induced by endogenous and environmental genotoxic stress. Here we show that short ssDNA traverses the nuclear membrane, but is drawn into the nucleus by binding to the DNA replication and repair factors RPA and Rad51. Knockdown of RPA and Rad51 enhances cytosolic leakage of ssDNA resulting in cGAS-dependent type I IFN activation. Mutations in the exonuclease TREX1 cause type I IFN-dependent autoinflammation and autoimmunity. We demonstrate that TREX1 is anchored within the outer nuclear membrane to ensure immediate degradation of ssDNA leaking into the cytosol. In TREX1-deficient fibroblasts, accumulating ssDNA causes exhaustion of RPA and Rad51 resulting in replication stress and activation of p53 and type I IFN. Thus, the ssDNA-binding capacity of RPA and Rad51 constitutes a cell intrinsic mechanism to protect the cytosol from self DNA.

## Abstract (web summary)

A central antiviral defence is immune recognition of cystolic DNA. Here the authors show that RPA and RAD51, in cooperation with TREX1, function to protect the cytosol from self-DNA.

## Graphical abstract

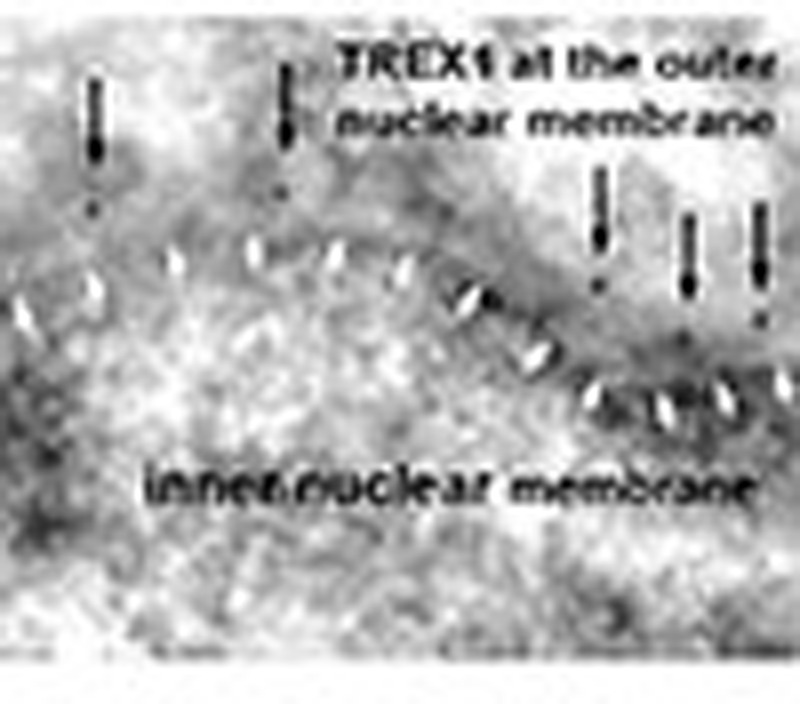

Activation of type I interferon (IFN) initiated by innate immune sensing of nucleic acids plays a key role in the pathogenesis of autoimmunity. Cytosolic RNA and DNA are sensed by pattern-recognition receptors such as RIG-I/MDA5 and cGAS, respectively^1^. As these sensors have only limited capacity to discriminate between self and non-self nucleic acids, the organism must be equipped with efficient means to avoid inappropriate immune activation through nucleic acids emanating from metabolic processes such as DNA damage repair. Reactive oxygen species and ultraviolet light continuously cause numerous DNA lesions most of which are efficiently repaired by the DNA repair machinery resulting in the excision of short single-stranded DNA (ssDNA) byproducts^2^. However, how the cell deals with this nuclear DNA waste is largely unknown.

TREX1 is the major cytosolic exonuclease in mammalian cells and acts preferentially on ssDNA^3^,^4^. Mutations in *TREX1* cause a spectrum of type I IFN-dependent autoinflammatory and autoimmune phenotypes including Aicardi–Goutières syndrome (AGS), familial chilblain lupus, retinal vasculopathy with cerebral leukodystrophy (RVCL) and systemic lupus erythematosus (SLE)^5^,^6^,^7^,^8^,^9^. AGS is also caused by mutations in the ribonuclease H2 complex^10^, the triphosphohydrolase SAMHD1 (ref. 11) and the RNA-editing enzyme ADAR^12^ highlighting the importance of the intracellular nucleic acid metabolism in the protection from autoimmunity.

*Trex1*^*−/−*^ mice develop type I IFN-mediated autoimmune disease initiated in non-hematopoietic cells and succumb to cardiac failure^13^,^14^. Type I IFN activation in TREX1-deficient mice was shown to be caused by cGAS-dependent sensing of cytosolic DNA^15^,^16^,^17^, yet the mechanisms underlying the formation of TREX1 substrates remain controversial. In *Trex1*^*−/−*^ mouse embryonic fibroblasts (MEF), accrual of cytosolic ssDNA has been attributed to aberrant DNA replication intermediates induced by Ataxia telangiectasia-mutated (ATM)-dependent checkpoint activation^18^. Conversely, autoimmunity in *Trex*^*−/−*^ mice was reported to be triggered by retroelement complementary DNA (cDNA) in the absence of checkpoint signalling^13^. In fibroblasts of AGS patients with RNase H2 or SAMHD1 deficiency, defective ribonucleotide excision repair or depletion of dNTP pools, respectively, cause chronic low-level DNA damage leading to constitutive activation of p53 and type I IFN^19^,^20^, raising the question as to how DNA damage signalling may be linked to type I IFN activation in TREX1 deficiency.

Here we report that short ssDNA arising within the nucleus is retained within the nuclear compartment by binding to the ssDNA-binding proteins replication protein A (RPA) and recombination protein A (Rad51) and establish that RPA and Rad51 depletion enhances cytosolic leakage of short ssDNA leading to type I IFN activation in a cGAS-dependent manner. Furthermore, we demonstrate that TREX1 is a tail-anchored protein inserted into the outer nuclear membrane to guard the cytosol from nuclear self DNA. In TREX1-deficient patient cells, accrual of ssDNA causes exhaustion of RPA and Rad51 resulting in replication stress and DNA damage checkpoint signalling alongside type I IFN activation. Thus, these findings delineate a novel mechanism that links pathways of DNA replication and repair with innate immune activation in the pathogenesis of autoimmunity.

## Results

### RPA and Rad51 prevent cytosolic leakage of short ssDNA

**Figure 1:**
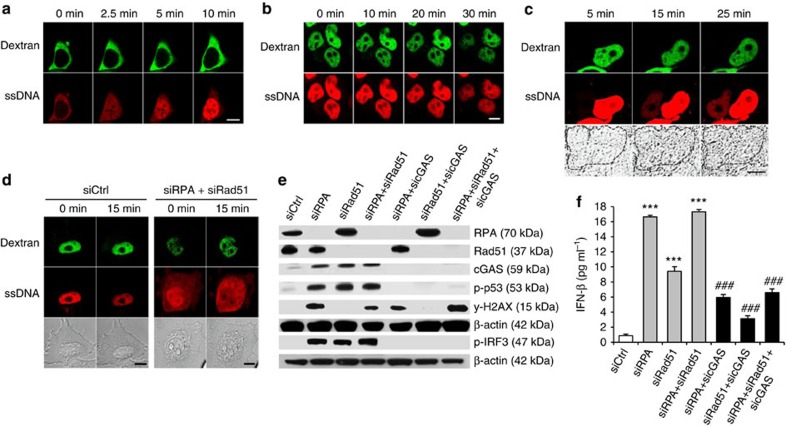
The ssDNA-binding of RPA and Rad51 prevents type I IFN activation caused by leakage of nuclear ssDNA into the cytosol. (**a**,**b**) The cytoplasm (**a**) or the nucleus (**b**) of HEK293T cells was microinjected with ssDNA647N (ssDNA, red) and FITC-dextran (dextran, green) as tracer. Images were taken at the indicated time points. Scale bar, 10 μM. (**c**) Representative image of a binuclear HEK cell. Fluorescence of ssDNA647N (ssDNA, red) microinjected into one nucleus, marked by FITC-dextran (dextran, green), is taken up by the non-injected adjacent nucleus. Scale bar, 10 μM. (**d**) ssDNA647N (ssDNA) microinjected into nuclei of HeLa cells with knockdown of RPA70 and Rad51 (siRPA+siRad51) leaks into the cytosol within 15 min, but remains nuclear in cells transfected with negative control siRNA (siCtrl). Co-injected FITC-dextran (green) marks the injected compartment. Scale bar, 10 μM. (**e**,**f**) Phosphorylation of IRF3 and p53 (**e**) as well as IFN-β induction (**f**) after knockdown of RPA70 and Rad51 (siRPA, siRad51) is suppressed by additional knockdown of cGAS. siCtrl, negative control siRNA. (**f**) Shown are the means of one experiment run in triplicates out of two independent experiments. Error bars, s.d. ***P<0.001 versus siCtrl. ^###^*P*<0.001 versus knockdown of RPA70, Rad51 or RPA70 and Rad51, respectively, by analysis of variance followed by Tukey' *post hoc* test.

To investigate the transit of short ssDNA across the nuclear membrane, we microinjected a 30-bp ATTO647N-labelled DNA oligonucleotide (ssDNA647N) into the cytoplasm or the nucleus of HEK293T cells. Microinjection into the cytoplasm resulted in rapid nuclear accumulation of the ssDNA647N oligonucleotide (Fig. 1a). In contrast, if ssDNA647N was microinjected into the nucleus, the fluorescent signal remained nuclear (Fig. 1b). Intriguingly, in cells with two nuclei, one of which was microinjected with ssDNA647N, the non-injected nucleus became fluorescent over time indicating leakage of the oligonucleotide into the cytosol and subsequent uptake by the non-injected nucleus (Fig. 1c and Supplementary Movie 1). We, therefore, hypothesized that short ssDNA, albeit capable of passively or actively crossing the nuclear membrane, is retained within the nucleus by binding to nuclear proteins.

DNA processing during replication and repair requires the ssDNA-binding proteins RPA and Rad51 to protect ssDNA stretches from nucleolytic degradation, to prevent reannealing and to coordinate assembly of processing factors. The RPA heterotrimer binds ssDNA with an occluded length of 30 nucleotides^21^, while Rad51 recombinase forms nucleofilaments on ssDNA binding^22^. We, therefore, examined whether knockdown of RPA and Rad51 would alter the subcellular distribution of the ssDNA647N oligonucleotide. Following microinjection of ssDNA647N into the nucleus of HeLa cells with small interfering RNA (siRNA)-induced knockdown of RPA and Rad51, the fluorescent signal spread throughout the cytosol, while it remained confined to the nucleus in cells treated with negative control siRNA (Fig. 1d) indicating that a reduction of the nuclear pools of RPA and Rad51 causes enhanced leakage of ssDNA into the cytosol.

### Depletion of RPA and Rad51 activates type I IFN

The nucleotidyl transferase cGAS is a potent sensor of cytosolic double-stranded DNA (dsDNA), but can also be activated by short ssDNA^23^,^24^,^25^. Given our observation that depletion of RPA and Rad51 results in enhanced cytosolic leakage of short ssDNA, we examined whether knockdown of RPA and Rad51 is sufficient to cause type I IFN activation. siRNA-induced depletion of RPA and Rad51 in HeLa cells led to IRF3 phosphorylation and type I IFN activation as shown by increased IFN-β production and up-regulation of IFN-stimulated genes (ISG) (Fig. 1e,f and Supplementary Fig. 1a). Type I IFN activation was markedly suppressed in cells with additional knockdown of cGAS indicating that ssDNA oozing out from the nucleus engages the cytosolic DNA sensor cGAS (Fig. 1e,f). Furthermore, analysis of transcriptome data retrieved from the GEO database revealed ISG upregulation in MEFs with short hairpin RNA-induced knockdown of RPA (accession number GSE38412) (ref. 26) and in human MCF-10A cells with short hairpin RNA-induced knockdown of Rad51 (accession number GSE54266) (ref. 27), respectively, consistent with a transcriptional signature due to spontaneous IFN production (Supplementary Fig. 1b,c).

Heterozygous loss-of-function mutations in the Rad51 paralogue *RAD51C* are a rare cause of breast and ovarian cancer^28^. Since Rad51 depletion enhances type I IFN signalling *in vitro*, we examined patients with breast or ovarian cancer due to *RAD51C* mutation for signs of autoimmunity. Intriguingly, three out of four patients were found to present with autoimmune features such as antinuclear antibodies, autoimmune thyroid disease or an IFN signature in blood (Supplementary Table 1). Taken together, these findings indicate that depletion of RPA and Rad51 promotes cytosolic accrual of ssDNA which triggers type I IFN signalling.

### Accumulation of ssDNA exhausts RPA and Rad51

**Figure 2:**
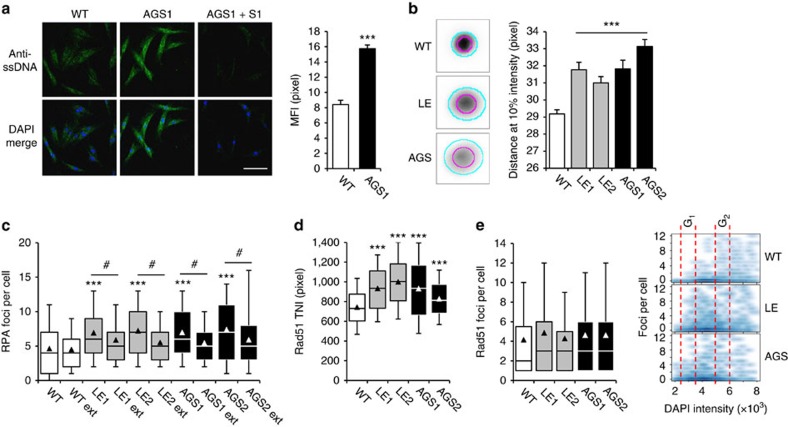
TREX1-deficient cells accumulate short ssDNA in the cytosol and nucleus and exhibit enhanced ssDNA-binding of RPA and Rad51. (**a**) Accumulation of ssDNA in the cytoplasm and nucleus of patient (AGS1) compared with wild-type (WT) cells visualized by immunostaining (anti-ssDNA, green, left). AGS1 cells pretreated with S1 nuclease (AGS1+S1) were stained as control. Nuclei are counterstained with DAPI (blue). Scale bar, 100 μm. Mean fluorescence intensities (MFI) are depicted on the right. Data are from at least three independent experiments. Error bars, s.e.m. ***P<0.001. (**b**) Perinuclear halo formation in TREX1-deficient (LE, AGS) and WT fibroblast nuclei. Indicated are the diameters of at 50% (magenta) and 10% (cyan) of the maximum intensities (left). Quantification of halo size at 10% maximum intensity (right). Shown are the means of two independent experiments for each patient (LE1, LE2, AGS1 and AGS2) and WT controls (*n*=2). Error bars, s.e.m. ***P<0.001. (**c**) Nuclear RPA foci formation without and after detergent extraction (ext). ***P<0.05 versus WT; ^#^*P*<0.001 versus non-extracted nuclei. (**d**) Total nuclear intensity (TNI) of Rad51. (**e**) Nuclear Rad51 foci in TREX1-deficient cells distribute more evenly throughout the cell cycle as shown by DNA (DAPI) content analysis. (**c**–**e**) WT controls (*n*=2). Box plots indicate the interquartile range (25–75%) from at least two independent experiments. Solid lines, median. Triangles, mean. Whiskers, 10–90th percentiles. *P<0.05; ***P<0.001 versus WT, Kruskal–Wallis test.

In TREX1 deficiency, type I IFN activation and autoimmunity result from immune recognition of unmetabolized cytosolic ssDNA. In line with previous findings in *Trex1*^*−/−*^ MEFs^18^, immunostaining of ssDNA in TREX1-deficient patient fibroblasts revealed a marked signal both within the cytosol and the nucleus, while ssDNA was barely detectable in wild-type cells. ssDNA was undetectable in TREX1-deficient cells pretreated with S1 nuclease confirming specific staining of ssDNA (Fig. 2a). To further define the size range of the accumulating ssDNA in patient cells, we employed the halo assay, which is based on the size-dependent diffusion of DNA fragments out of agarose-embedded nuclei^29^. Transfection of fibroblasts with increasing amounts of a 45-bp oligonucleotide resulted in increasing halo diameters in a dose-dependent manner (Supplementary Fig. 2). We next assessed halo formation in TREX1-deficient fibroblasts and observed larger halo diameters compared with wild-type cells indicating enhanced levels of short DNA molecules within the nucleus (Fig. 2b).

To explore whether ssDNA accumulating in TREX1-deficient fibroblasts is bound by RPA and Rad51, we performed automated high-content imaging. Unlike wild-type cells, patient fibroblasts exhibited an increased number of nuclear RPA foci, which decreased on extraction of cells with detergent (Fig. 2c), indicating the presence of an ssDNA-bound RPA fraction not associated with chromosomal DNA. To validate this further, we measured the mobility of RPA complexes in living cells transfected with GFP-RPA32 using raster image correlation spectroscopy (Supplementary Fig. 3). In non S-phase cells, the mean diffusion coefficient of GFP-RPA32 was significantly lower in AGS cells (8.2±1.7 μm^2^ s^−1^) than in wild-type cells (11.9±2.8 μm^2^ s^−1^) indicating that RPA is part of a larger complex in AGS cells. In S-phase cells, RPA is present in two forms, free RPA and focal RPA engaged in replication. Again the AGS cells exhibited a reduced RPA diffusion compared to wild type cells (2.2±0.9 versus 3.6±1.0 μm^2^ s^−1^), while the RPA fraction engaged in replication foci exhibited nearly no mobility in both patient and wild-type cells (0.3±0.6 versus 0.3±0.3 μm^2^ s^−1^). In addition, we quantified the amount of ssDNA, RPA and Rad51 using high-resolution imaging following immunofluorescence (Supplementary Fig. 4). This confirmed markedly enhanced levels of ssDNA in the cytoplasm of AGS cells compared with wild-type controls. Moreover, we observed an increased co-localization between ssDNA and RPA or Rad51, respectively, in the cytoplasm of TREX1-deficient patient fibroblasts, while there was no difference in co-localization within the nucleus. Finally, we determined the intensity ratio between ssDNA and RPA or Rad51, respectively, and found an approximately twofold increase in AGS cells compared with wild-type cells suggesting an exhaustion of cellular RPA and RAD51 pools (Supplementary Fig. 4).

Furthermore, TREX1-deficient fibroblasts also showed an increased nuclear Rad51 intensity compared with wild-type cells consistent with enhanced formation of Rad51 complexes (Fig. 2d). Although TREX1-deficient cells did not harbour more Rad51 foci than wild-type cells, foci were more evenly distributed throughout the cell cycle as shown by DNA content analysis (Fig. 2e and Supplementary Fig. 5) indicating Rad51 nucleofilament formation independent of homologous recombination repair in G_2_. Thus, increased formation of ssDNA in TREX1-deficient patient cells leads to enhanced occupation of RPA and Rad51 by ssDNA exhausting the nuclear pool of RPA and Rad51.

### TREX1 is a tail-anchored protein

**Figure 3:**
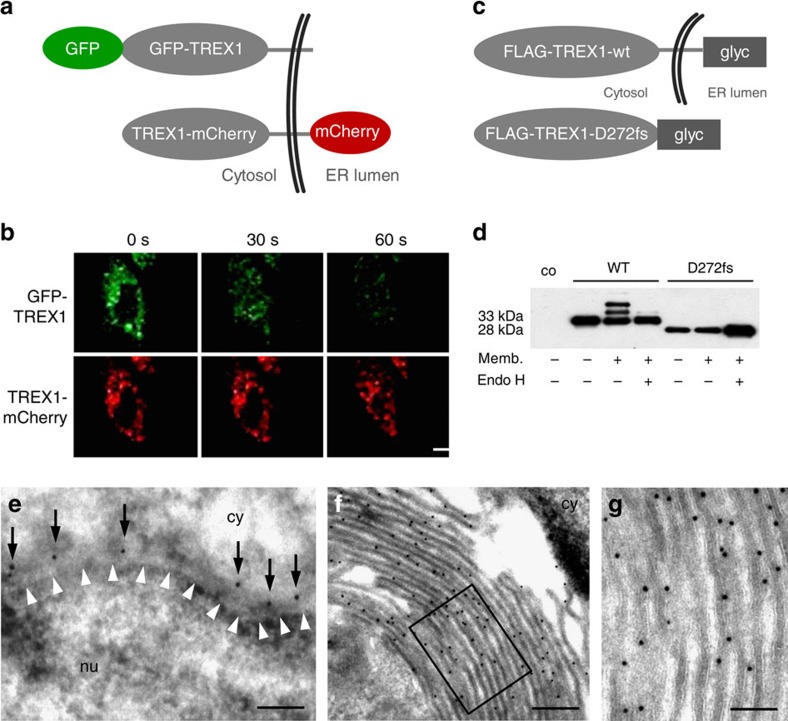
TREX1 is a tail-anchored protein. (**a**) Schematic of fluorescently tagged TREX1 constructs used for fluorescence protease protection assay. (**b**) Confocal images of living HeLa cells co-expressing GFP-TREX1 and mCherry-TREX1. Proteinase K treatment after selective plasma membrane permeabilization leads to rapid dissipation of cytosolic GFP fluorescence, while mCherry resists proteolysis. Scale bar, 20 μm. (**c**) Schematic of TREX1 constructs containing a C-terminal bovine opsin fragment with two N-linked glycosylation sites (glyc) used for glycosylation reporter assay. (**d**) *In vitro* translated WT FLAG-TREX1-wt-glyc, but not mutant FLAG-TREX1-D272fs-glyc lacking the transmembrane domain, is glycosylated in the presence of microsomal membranes (memb.) as visualized by anti-FLAG immunoblotting. Glycosylation of WT TREX1 is removed by endoglycosidase H (endo H). (**e**) Electron microscopy of immunogold-labelled GFP-TREX1 in HeLa cells shows expression of TREX1 (black dots marked by arrows) along the outer nuclear membrane and the cytosolic site of the ER membrane. White arrowheads indicate the inner nuclear membrane. Scale bar, 200 nm. (**f**) Formation of organized smooth ER consisting of lamellar stacks, branching tubules or whorls in cells overexpressing GFP-TREX1. Scale bar, 200 nm. (**g**) Magnification of the inset in (**f**) shows an even distribution of GFP-TREX1 at the extraluminal site of the ER. Scale bar, 100 nm. co, no lysate control; cy, cytoplasm; nu, nucleus.

TREX1 associates with the endoplasmic reticulum (ER) that requires the C terminus containing a predicted transmembrane helix. ER localization is functionally indispensable as truncating TREX1 mutations that preserve nuclease activity, but abrogate ER targeting cause RVCL and SLE^7^,^8^. Although the C terminus of TREX1 is sufficient to confer ER localization, it was suggested that TREX1 is not a transmembrane protein^13^. We, therefore, investigated the membrane topology of TREX1 by fluorescence protease protection assay^30^. Treatment of permeabilized HeLa cells co-expressing GFP-TREX1 and TREX1-mCherry with proteinase K led to rapid loss of green fluorescent protein (GFP) fluorescence, while the C-terminal mCherry-tag was protected from proteolysis (Fig. 3a,b) indicating membrane integration of TREX1. We excluded different bleaching properties of GFP and mCherry by showing similar fluorescence decay curves (Supplementary Fig. 6). To further confirm transmembrane orientation of TREX1, we determined that glycosylation of *in vitro* translated wild-type TREX1 (FLAG-TREX1-wt-glyc) and the truncation mutant D272fs (FLAG-TREX1-D272fs-glyc), respectively, fused to a bovine opsin fragment containing two glycosylation sites at the C-terminal end (Fig. 3c,d). Consistent with orientation of the C terminus into the ER lumen, FLAG-TREX1-wt-glyc was glycosylated in the presence of microsomal membranes as shown by the appearance of two additional bands of higher molecular weight that disappeared after treatment with endoglycosidase H (Fig. 3d). In contrast, FLAG-TREX1-D272fs-glyc, which lacks the transmembrane helix and is not targeted to the ER^7^,^8^, was not glycosylated (Fig. 3d). Electron microscopy of immunogold-labelled GFP-TREX1 in HeLa cells revealed TREX1 localization along the outer nuclear membrane and the cytosolic site of the ER membrane (Fig. 3e) as well as formation of organized smooth ER, highly organized structures typically induced by overexpression of ER membrane proteins (Fig. 3f,g)^31^. Thus, TREX1 is a tail-anchored protein with the nuclease domain facing the cytosol.

### Reduced cytosolic exonuclease activity in patient cells

**Figure 4:**
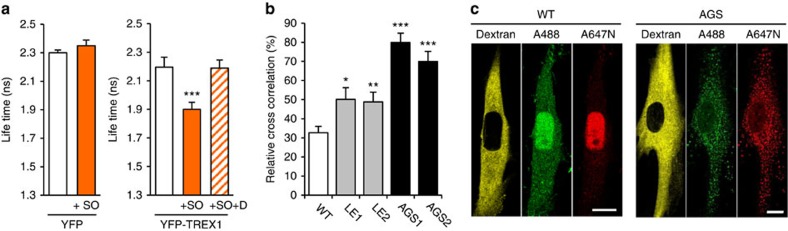
*In situ* biochemistry of TREX1-deficient cells. (**a**) Interaction of YFP-TREX1 with endogenous cytosolic nucleic acids in HeLa cells analysed by FLIM-FRET. The fluorescence lifetime of YFP-TREX1 (right), but not of YFP alone (left), decreases significantly in the presence of Sytox Orange (+SO). The lifetime reduction of YFP-TREX1 is abolished in cells pretreated with DNase I (+SO+D). At least ten cells were measured per experiment. Means and s.d. of three independent experiments. ***P<0.001. (**b**) FCCS of HeLa cells microinjected with dsDNA488_647N. Mean cross-correlation and s.e.m. of three independent experiments measured at t<60 min. (WT, *n*=2). *P<0.05; **P<0.01; ***P<0.001. (**c**) Representative FCCS images. ATTO488 (A488, green), ATTO647N (A647N, red). Rhodamine B-dextran (yellow) marks the microinjected cytosol. Scale bar, 10 μm.

To gain further insight into the *in situ* biochemistry of TREX1, we first examined the interaction of YFP-TREX1 with endogenous cytosolic DNA stained with the fluorescent nucleic acid stain Sytox Orange by fluorescence lifetime imaging microscopy. The lifetime of YFP-TREX1, but not YFP alone, significantly decreased in the presence of Sytox Orange (Fig. 4a). Preincubation of cells with DNase I before Sytox Orange staining abolished the lifetime reduction of YFP-TREX1 confirming that TREX1 interacts with endogenous cytosolic DNA *in situ* (Fig. 4a). We next analysed the 3′-exonuclease activity in living cells by fluorescent cross-correlation spectroscopy using a dual-labelled 30 bp dsDNA oligonucleotide with 3′-overhangs (dsDNA488_647N) as substrate. In wild-type fibroblasts, microinjection of dsDNA488_647N into the cytoplasm led to a decline of cross-correlation amplitudes by 70% within few minutes and to spreading of the green and red fluorescence throughout the cell indicating rapid nucleolytic degradation (Fig. 4b,c). In contrast, cross-correlation decayed significantly slower in fibroblasts from an AGS patient consistent with decreased cytosolic exonuclease activity (Fig. 4b). Moreover, the nucleus did not become fluorescent over the course of 60 min (Fig. 4c) suggesting that the undegraded dsDNA oligonucleotide was not capable of crossing the nuclear membrane or that the nuclear ssDNA-binding capacity was already exhausted in AGS patient cells.

### DNA damage signalling and type I IFN activation in patient cells

**Figure 5:**
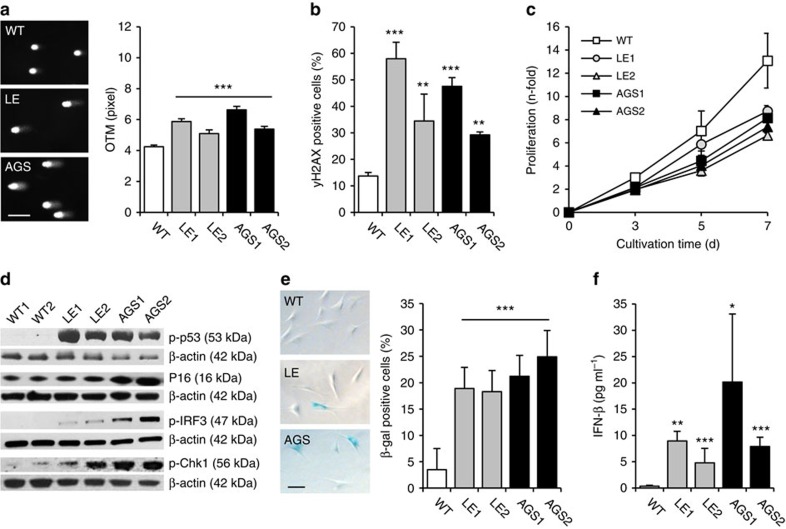
Constitutive DNA damage and type I interferon activation in TREX1-deficient cells. (**a**) Alkaline single-cell electrophoresis depicting DNA damage in patient fibroblasts (LE, AGS) as shown by formation of longer comets with deformed nuclei compared with wild type cells (WT). Scale bar, 100 μm (left). The Olive tail moment (OTM) corresponds to the amount of DNA fragmentation (right). Means and s.e.m. of two independent experiments for each patient (LE1, LE2, AGS1, AGS2) and WT control cell lines (WT, *n*=2). ***P<0.001. (**b**) Increased pan-nuclear γH2AX-staining in TREX1-deficient fibroblasts determined by flow cytometry. Means and s.d. of at least three independent experiments for each patient (LE1, LE2, AGS1, AGS2) and WT controls (WT, *n*=5). **P<0.01; ***P<0.001. (**c**) Growth curves of TREX1-deficient fibroblasts (LE1, LE2, AGS1, AGS2) and WT cells (*n*=5). *P<0.05 (LE1) and **P<0.01 (LE2, AGS1, AGS2) versus WT at day 7. (**d**) Immunoblot analysis of unstressed patient fibroblasts (LE1, LE2, AGS1, AGS2) showing activation of p53 (p-p53; Ser15), p16, Chk1 (p-Chk1; Ser345) as well as IRF3 (p-IRF3). β-actin was probed as a loading control. (**e**) Senescent phenotype of patient fibroblasts (LE1, LE2, AGS1, AGS2) as shown by an increased percentage of β-galactosidase-positive cells (WT, *n*=4; left) and representative images of senescent cells (right). Scale bar, 100 μm. Means and s.d. of at least three independent experiments. *P<0.05; **P<0.01; ***P<0.001. (**f**) IFN-β secretion over 24 h. (WT, *n*=4). Means and s.e.m. of five independent experiments. *P<0.05; **P<0.01; ***P<0.001 versus WT.

RPA-coated ssDNA at sites of DNA damage initiates checkpoint signalling^32^, while global exhaustion of nuclear RPA pools causes replication fork stalling that promotes DNA damage^33^. Rad51 plays an important role in DNA damage tolerance as it participates in replicative repair of single-strand DNA lesions and replication fork reversal^34^,^35^. Given enhanced ssDNA binding of RPA and Rad51 in patient cells, we hypothesized that depletion of unbound RPA and Rad51 could cause secondary DNA damage. We, therefore, investigated the overall genome integrity of fibroblast from AGS and SLE patients by alkaline single-cell gel electrophoresis, which detects alkali-labile sites such as single-strand DNA breaks, abasic sites or incomplete excision repair sites, as well as stalled replication forks. In the absence of exogenous genotoxic stress, patient fibroblasts harboured significantly more DNA damage compared with wild-type cells as shown by the formation of longer comets (Fig. 5a). Co-staining of phosphorylated histone H2AX (γH2AX) and p53-binding protein 1 (53BP1) did not reveal an increase in DNA double-strand breaks in TREX1-deficient cells (Supplementary Fig. 7e). However, the number of cells with diffuse pan-nuclear γH2AX staining was markedly enhanced consistent with single-strand DNA damage (Fig. 5b and Supplementary Fig. 7f).

**Figure 6:**
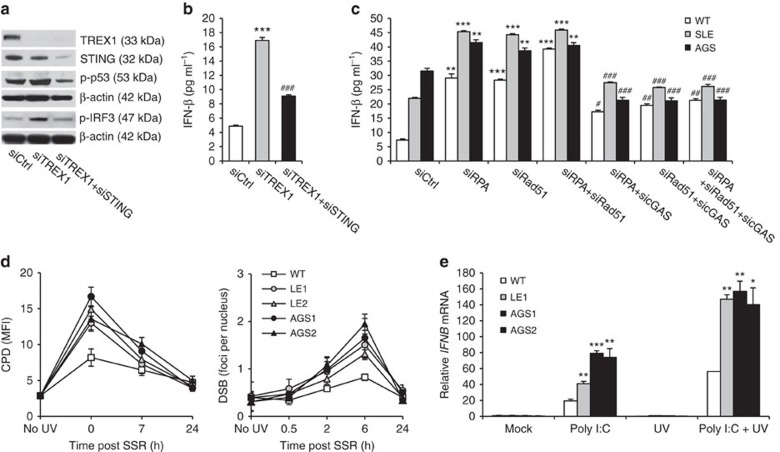
TREX1-deficient cells are DNA repair-proficient, but more sensitive to type I interferon-dependent stimuli. (**a**,**b**) Knockdown of TREX1 (siTREX1) in HeLa cells causes phosphorylation of p53 and IRF3 along with induction of IFN-β. This is abrogated in cells with additional knockdown of STING (siTREX+siSTING). siCtrl, negative control siRNA. ***P<0.001 versus siCtrl; ^###^*P*<0.001 versus siTREX1. (**c**) Constitutive activation of IFN-β in TREX1-deficient fibroblasts (SLE, AGS) is further enhanced by siRNA-induced knockdown of RPA70 (siRPA) or Rad51 (siRad51) or both (siRPA+siRad51). This is suppressed by additional knockdown of cGAS (sicGAS). Shown are the means of one experiment run in triplicates out of two independent experiments. Error bars, s.d. *P<0.001 versus siCtrl. ^#^*P*<0.001 versus knockdown of RPA70, Rad51 or RPA70 and Rad51, respectively, by analysis of variance followed by Tukey's *post hoc* test. (**d**) Repair kinetics of CPDs (left) and DSBs (right) in response to solar-simulated radiation (SSR). Means and s.e.m. of at least three independent experiments for each patient and wild-type control cell lines (WT, *n*=5). *P<0.05 (LE1, LE2) and **P<0.01 (AGS1, AGS2) immediately after ultraviolet exposure for CPDs, *P<0.05 (LE1, LE2) and **P<0.01 (AGS1, AGS2) at 6 h for DSBs. Although TREX1-deficient cells react more sensitive to SSR than WT cells, both DNA lesions are efficiently repaired within 24 h post irradiation. (**e**) *IFNB* upregulation in patient fibroblasts challenged with poly(I:C) and ultraviolet C irradiation. (WT, *n*=2). Means and s.e.m. of five independent experiments. *P<0.05; **P<0.01; ***P<0.001 versus WT. *P<0.05 (AGS2); **P<0.01 (LE1); ***P<0.001 (LE2, AGS1) for poly(I:C) versus poly(I:C) plus ultraviolet C. Mann–Whitney *U*-test.

To further investigate the consequences of chronic DNA damage in TREX1-deficient fibroblasts, we examined their cellular phenotype. Patient cells proliferated slower than wild-type cells due to defective G_1_/S transition (Fig. 5c and Supplementary Fig. 7a,b). This was accompanied by induction of the tumour suppressors p53, p16 and p21 as well as the DNA damage signalling kinases ATM and Chk1 (Fig. 5d, and Supplementary Fig. 7c,d). In addition, patient cells displayed a marked increase of senescence-associated β-galactosidase-positive cells with an enlarged morphology (Fig. 5e). Simultaneously, patient fibroblasts exhibited constitutive IRF3 phosphorylation and transactivation of an IFN-β reporter (Fig. 5d,f). Transcriptional profiling confirmed upregulation of both p53-dependent and ISG (Supplementary Fig. 8). Notably, siRNA-induced knockdown of TREX1 in HeLa cells was sufficient to induce both activation of p53 and type I IFN, which was abrogated in cells with additional knockdown of STING, a key signalling molecule of the cytosolic DNA sensing pathway (Fig. 6a,b). In contrast, constitutive activation of type I IFN and DNA damage signalling could be rescued in fibroblasts of an AGS patient by overexpression of wild type TREX1 (Supplementary Fig. 9). Moreover, in TREX1-deficient patient fibroblasts, knockdown of RPA and Rad51 led to a further increase in type I IFN production that was markedly suppressed by additional knockdown of cGAS (Fig. 6c), suggesting that RPA, Rad51 and TREX1 function along the same pathway to prevent accumulation of nuclear DNA within the cytosolic compartment. Binding of unmetabolized ssDNA by RPA and Rad51 causes chronic consumption of RPA and Rad51, which results in genomic instability. Collectively, these findings delineate a cell-autonomous link between a p53-dependent DNA damage response and cGAS-STING-IRF3-dependent innate immune activation in TREX1 deficiency.

### TREX1-deficient cells are DNA repair-proficient

The DNA damage phenotype of TREX1-deficient cells and the finding that the AGS-causing genes, *RNASEH2* and *SAMHD1*, function in pathways of DNA replication and repair^19^,^20^,^36^,^37^, suggested that TREX1 notwithstanding its cytosolic localization might be involved in DNA replication or repair. We, therefore, investigated the role of TREX1 in processes of DNA replication and repair in more detail. First, we examined distribution of GFP-tagged TREX1 throughout the cell cycle in HeLa cells stably expressing mCherry-tagged proliferating-cell nuclear antigen, a central component of the replication machinery. While GFP-RNASEH2B, a subunit of the genome surveillance enzyme RNase H2, was recruited to replication foci during S-phase, GFP-TREX1 did not associate with replication sites (Supplementary Fig. 10a,b). Furthermore, TREX1 was not recruited to sites of hydroxurea-induced DNA damage as shown by co-staining of γH2AX and 53BP1 (Supplementary Fig. 10c). Second, we examined the effects of genotoxic stress in patient fibroblasts. We measured formation of cyclobutane pyrimidine dimers (CPDs), the most abundant primary photolesions, and of secondary DNA double-strand breaks in response to solar-simulated ultraviolet radiation. Irradiation with ultraviolet light led to a slightly increased formation of CPDs and DNA double-strand breaks in patient fibroblasts compared with wild-type cells (Fig. 6d), suggesting that pre-existing genomic instability or the exhaustion of essential DNA repair factors renders TREX1-deficient cells more vulnerable to exogenous genotoxic stress. However, both lesions were equally efficiently repaired in patient and wild-type fibroblasts (Fig. 6d). Thus, TREX1-deficient cells are proficient in pathways of nucleotide excision repair and DNA double-strand break repair.

Like fibroblasts of AGS patients with RNase H2 or SAMHD1 deficiency^19^,^20^, TREX1-deficient patient cell exhibit constitutive activation of type I IFN signalling. We, therefore, hypothesized that a cell intrinsic activation of ISGs could render TREX1-deficient cells more sensitive to IFN-dependent stimuli^38^. To further explore this, we investigated *IFNB* expression in fibroblasts challenged with poly(I:C), a synthetic dsRNA and viral mimic. TREX1-deficient cells responded with a stronger upregulation of *IFNB* than wild-type cells (Fig. 6e). This effect was even more pronounced in cells additionally exposed to environmental genotoxic stress in the form of ultraviolet light, a well-known trigger of SLE flares, suggesting that ultraviolet light could promote type I IFN activation in TREX1-deficient cells by enhancing formation of ssDNA byproducts resulting from repair of DNA photodamage.

## Discussion

In this study, we delineate a role of the ssDNA-binding capacity of RPA and Rad51 in the protection of the cytosolic compartment from self DNA emanating from nuclear DNA metabolism. Cells are constantly exposed to genotoxic influences derived from endogenous metabolic byproducts or environmental exposures resulting in tens of thousands of DNA lesions per cell each day, most of which are immediately repaired by DNA repair mechanisms with remarkable efficiency^2^. Nucleotide excision repair, which removes bulky helix-distorting DNA lesions such as ultraviolet light-induced CPDs as well as oxidative DNA lesions, results in the excision of short ssDNA byproducts^39^,^40^. Consequently, the cell is challenged by an enormous load of DNA metabolites that need to be properly disposed of to avoid activation of an immune response through recognition by innate immune DNA sensors. While the byproducts of DNA repair are thought to be degraded by nucleases, their intracellular fate remains poorly understood.

The ssDNA-binding proteins RPA and Rad51 play essential roles in pathways of DNA replication, recombination and repair and are abundantly expressed in the nucleus. RPA is a heterotrimeric complex composed of the RPA70, RPA32 and RPA14 subunits, which binds to ssDNA through multiple oligonucleotide/oligosaccharide-binding fold domains^21^. Rad51 on the other hand forms nucleoprotein filaments consisting of multiple ssDNA-bound Rad51 molecules^22^. RPA participates in DNA replication by enhancing the assembly of DNA polymerases. In addition, both RPA and Rad51 are mobilized to sites of DNA damage, where they protect ssDNA stretches from nucleolytic damage and coordinate the recruitment and exchange of processing factors of a number of DNA repair pathways, in particular during nucleotide excision repair and homologous recombination repair^41^,^42^.

Short DNA fragments microinjected into living cells were shown to move between the nucleus and the cytoplasm^43^,^44^ suggesting that they are either actively exported/imported or freely diffusing through the nuclear pore complexes. Indeed, we demonstrate that short ssDNA microinjected into living cells traverses the nuclear membrane in both directions, but is preferentially drawn into the nucleus. This is illustrated by the observation that ssDNA molecules oozing out from the microinjected nucleus of a binuclear cell are taken up by the non-injected nucleus of the same cell. Nuclear retention of ssDNA is mediated by RPA and Rad51, which bind ssDNA with high affinity. Thus, depletion of RPA and Rad51 causes enhanced cytosolic leakage of exogenous ssDNA microinjected into the nucleus, while endogenous ssDNA accumulating in TREX1-deficient patient cells is bound by RPA and Rad51 as shown by an increased fraction of ssDNA-bound RPA not associated with chromatin as well as enhanced formation of Rad51 nucleoprotein filaments in the absence of exogenous genotoxic stress. Intriguingly, depletion of RPA and Rad51 in HeLa cells is sufficient to induce type I IFN activation in a cGAS-dependent manner indicating that ssDNA that has escaped the nuclear compartment is recognized by the cytosolic DNA sensor cGAS. Moreover, constitutive type I IFN activation in TREX1-deficient patient cells is further augmented by depletion of RPA and Rad51 suggesting that TREX1 acts in concert with RPA and Rad51 to guard the cytosol from nuclear self DNA.

TREX1 is targeted to the ER within the perinuclear region, which is mediated by a C-terminal hydrophobic domain that is not required for catalytic activity. Notably, C-terminal deletion mutations that impede ER targeting of TREX1 cause the autoimmune disorders RVCL and SLE^7^,^8^ underpinning a pivotal role of the subcellular localization of TREX1. Indeed, we demonstrate that TREX1 is a tail-anchored protein that integrates within the ER membrane and the outer nuclear membrane via the C-terminal hydrophobic domain with the nuclease domain facing the cytosol. Thus, TREX1 functions as a sentinel nuclease patrolling the border of the nuclear compartment to ensure immediate degradation of ssDNA metabolites leaking out of the nucleus before they can be accessed by the cytosolic DNA sensing machinery.

Recognition of DNA damage by RPA-ssDNA complex formation constitutes a key signal in the activation of ATR kinase-dependent cell cycle checkpoint signalling to protect and repair stalled replication forks^32^. Notably, an excess of ssDNA caused by unscheduled origin firing was recently shown to exhaust the nuclear pool of RPA accelerating replication fork breakage^33^. Likewise, Rad51 recruitment to replication forks is a prerequisite for DNA damage tolerance pathways such as replicative repair of single-strand DNA lesions and replication fork reversal^34^,^35^. Hence, augmented binding of ssDNA to RPA and Rad51 in TREX1-deficient cells could promote exhaustion of the nuclear ssDNA-binding capacity leading to chronic checkpoint activation and replication stress through unscheduled engagement of DNA replication/repair pathways. Consistent with this, TREX1-deficient patient fibroblasts exhibit chronic low-level single-strand DNA damage as indicated by an increased pan-nuclear H2AX phosphorylation, checkpoint signalling and a p53-dependent DNA damage response leading to senescence.

A direct role of TREX1 in DNA repair is largely excluded by its cytosolic location and the observation that TREX1-deficient fibroblasts proficiently repair CPDs and DNA double-strand breaks. Consequently, efficient DNA repair in TREX1-deficent cells enhances nuclear formation of ssDNA fragments further contributing to exhaustion of RPA and Rad51. ssDNA that has leaked into the cytosol and is not removed by TREX1 then act as danger-associated molecular pattern (Supplementary Fig. 11). Interestingly, DNA containing 8-oxoguanine, the most common oxidative DNA lesion, was shown to act as a damage-associated molecular pattern^45^, further linking the DNA damage response to innate immune activation.

Autoimmunity in patients with AGS and SLE is characterized by activation of type I IFN signalling^46^,^47^,^48^,^49^,^50^. In agreement with this, we demonstrate induction of ISGs in TREX1-deficient fibroblasts besides upregulation of DNA damage response genes. Notably, ISG activation occurred in cultured primary patient fibroblasts not exposed to type I IFN-secreting immune cells confirming a cell autonomous mechanism underlying innate immune activation. Interestingly, a STING/TBK1/IRF3-dependent ISG induction in *Trex1*^−/−^ MEFs is associated with increased lysosomal biogenesis^51^, a phenotype typically observed in senescent cells. Moreover, we demonstrate enhanced *IFNB* upregulation in patient cells in response to poly(I:C) and ultraviolet indicating that constitutive ISG upregulation has a priming effect that renders cells more sensitive to type I IFN-dependent stimuli^38^. Sunlight, a common trigger of SLE flares, induces primarily CPDs, which are repaired by nucleotide excision repair. Thus, ultraviolet light could create a vicious cycle that fuels and sustains autoimmunity in TREX1 deficiency (Supplementary Fig. 11). Chronic low-level DNA damage and enhanced sensitivity to genotoxic stress also suggest that patients with *TREX1* mutations may have an increased cancer risk, like patients with *SAMHD1* mutations^52^. Moreover, *Trex1*^*−/−*^ mice were reported to develop carcinogen-induced skin tumours in a STING-dependent manner^53^. It is of note, in this context, that lupus patient LE1 developed two independent malignancies and a precancerous lesion (Supplementary Note 1).

TREX1 most likely guards the cytosol against ssDNA of any source including viral DNA. *Trex1*^*−/−*^ MEFs were reported to accumulate ssDNA derived from retroelements^13^, although an increased retroelement activity could not be observed in macrophages or dendritic cells of *Trex1*^*−/−*^ mice^53^. On the other hand, TREX1 was shown to facilitate HIV-1 infection by degrading non-productive reverse transcripts of the HIV-1 RNA genome, thereby suppressing a STING-dependent antiviral response^54^. However, as an increased retroelement activity does not explain chronic DNA damage signalling observed in TREX1-deficient patient cells, the pathogenetic role of retroelement-derived DNA in human TREX1 deficiency remains unclear.

Our findings reveal a molecular framework in which the exonuclease TREX1 and the DNA replication and repair factors RPA and Rad51 act together in the disposal of nuclear DNA waste. Interestingly, the physiological role of the TREX1 homologue TREX2, which lacks the transmembrane domain and is expressed in the nucleus, is unknown^4^. It is therefore tempting to speculate that TREX2 might function in the degradation of such DNA metabolites within the nucleus. Further studies are required to test this idea. In conclusion, our findings establish an unanticipated role of the nuclear ssDNA-binding capacity of RPA and Rad51 as an innate defence against self DNA and provide novel mechanistic insight into pathways underlying nucleic acid-driven autoinflammation and autoimmunity.

## Methods

### Cell lines and plasmids

HeLa cells (ATCC), HEK293T cells (ATCC) and fibroblasts were cultured in DMEM supplemented with 10% FCS, 2 mM L-glutamine and 1% antibiotics–antimycotics. Passage-matched (passages 4–11) cells were used in all experiments. Human primary fibroblasts were derived from skin biopsies with written informed consent from patients or their parents. The study was approved by the ethics committee of the Medical Faculty, TU Dresden. The human TREX1-cDNA was cloned into pEGFP-C1 (GFP-TREX1) and pEGFP-N1 (TREX1-GFP). GFP was replaced with mCherry or YFP using *Age*I and *Bsr*GI. Mutations were introduced by site-directed mutagenesis (QuikChange Lightning, Agilent) or cDNA cloning from patient cells. FLAG-TREX1-glyc constructs were cloned into pCDNA3 by introducing an N-terminal FLAG tag and a fragment coding for the first 19 amino acids of bovine opsin containing two N-linked glycosylation sites (NH2-MNGTEGPNFYVPFSNKTVD-COOH, glycosylation sites are underlined)^55^ to the C terminus of wild-type or mutant TREX1. All constructs were verified by sequencing.

### Fluorescence protection assay

Fluorescence protection assay was performed as follows^30^: Briefly, HeLa cells were grown on chamber slides (Lab-Tek II, Nalge-Nunc) and transfected with 120 ng of each GFP-TREX1 and TREX1-mCherry plasmids using FuGENE HD (Roche). Cells were washed with PBS 48 h post transfection and placed on a confocal microscope stage. After taking the first image (pre-permeabilization state), 0.03% Triton X-100 in PBS was added for 30 s and a second image was taken to capture the post-permeabilization state. Following three washes with PBS, 50 μg ml^−1^ proteinase K was added and the disappearance of the fluorescence signal was documented in consecutive images taken at 30 s intervals. Microscopy was carried out on a Leica TCS SP5 microscope using a × 40 1.25–0.75 oil objective. For the detection of GFP and mCherry, an argon multiline laser (excitation 488 nm; emission 498–584 nm) and a HeNe laser (excitation 594 nm; emission 605–700 nm), respectively, were used.

### Glycosylation reporter assay

Templates for *in vitro* transcription/translation were generated by PCR amplification from FLAG-TREX1-glyc plasmids using a T7 promoter-containing sense primer. FLAG-TREX1-glyc proteins were generated using the TnT Quick Coupled Transcription/Translation System (Promega) either in absence or presence of 2 μl microsomal membranes. One aliquot of each reaction was treated with endoglycosidase H for 1 h at 37 °C before SDS–PAGE and western blot using anti-FLAG antibody (Sigma).

### Cell proliferation and cell cycle analysis

Fibroblasts were seeded at a density of 2 × 10^5^ cells per flask and counted at indicated time points using a Neubauer chamber. For DNA content analysis, fibroblasts were synchronized by serum starvation for 24 h. At the indicated time points after cultivation in complete growth medium, cells were collected, fixed and stained with propidium iodide. For BrdU labelling, synchronized cells were released at a ratio of 1:4 into complete growth medium containing 10 μM BrdU (BrdU Flow Kit, BD Pharmingen) and stained with 7-aminoactinomycin D (7-AAD) at 24 and 48 h of cultivation. For staining of p21 and γH2AX, trypsinized fibroblasts were fixed in methanol (−20 °C), permeabilized with 0.1% Tween and blocked in 1% BSA. For staining of pATM, cells were fixed in 4% fomaldehyde and permeabilized with 0.1% saponin. Cells were then incubated with anti-p21 (clone EA10, Abcam; 1:10), anti-γH2AX (Ser139, clone 2F3, Novus Biologicals; 1:500) or anti-pATM (Ser1981, abcam, 1:500) followed by incubation with Alexa Fluor 488-conjugated goat-anti-mouse-IgG (Molecular Probes). Flow cytometry was performed on a FACSCalibur (Becton Dickinson).

### RNA interference

HeLa cells were transfected with TREX1 siRNA (s229446), RPA70 siRNA 1 (s12127), RAD51 siRNA 1 (s11734), STING siRNA (s50644), cGAS siRNA (s41748) and control siRNA (Negative Control #1 siRNA) using Oligofectamine (Invitrogen). siRNAs were from Ambion. Lysates were prepared 72 h after transfection for western blot analysis and IFN-β reporter assay (Supplementary Methods). Images have been cropped for presentation. Full-size images are presented in Supplementary Fig. 12.

### Ultraviolet exposure

For determination of DNA repair kinetics, fibroblasts were grown on coverslips, washed with PBS and exposed to solar-simulated ultraviolet radiation using an Arimed B lamp at a radiation dosage of 20 J m^−2^ (DNA double-strand breaks) or 100 J m^−2^ (CPDs). At the indicated time points post irradiation, cells were fixed and stained for DSBs or CPDs. *IFNB* expression was determined in fibroblasts grown in six-well plates following exposure to ultraviolet C-light (254 nm, Hg-low pressure lamp) at a radiation dosage of 30 J m^−2^. Before ultraviolet C irradiation, cells were either left untreated or incubated with 100 μg ml^−1^ poly(I:C) (InvivoGen) for 3 h. Slides were then washed with PBS and fresh medium added. 4 h post irradiation, cells were processed for RNA isolation.

### Quantitative real-time reverse transcription–PCR

Total RNA from fibroblasts was extracted with the RNeasy Mini Kit (Qiagen) followed by DNase I digestion. Expression of *IFNB* and ISGs was determined by quantitative real-time reverse transcription–PCR using Taqman Universal PCR Master Mix (Applied Biosystems) on an ABI7300 and normalized to GAPDH expression. Each experiment was run triplicates. *IFI44L*, *IFIT1*, *ISG15* and *RSAD2* gene expression was determined using a pre-designed TaqMan Gene Expression Assay (Applied Biosystems). For oligonucleotides (Eurofins MWG Operon) used for quantitative real-time reverse transcription–PCR see Supplementary Table 2.

### Statistical analysis

Statistical significance was determined by two-tailed Student's *t*-test, Mann–Whitney *U*-test or analysis of variance followed by Tukey's *post hoc* test. Values of *P*<0.05 were considered statistically significant. Data are represented as means±s.d. or s.e.m. as indicated.

### Data availability

RNA sequencing data generated in this study were submitted to the Gene Expression Omnibus (GEO) database under accession number GSE59233.

## Additional information

**Accession codes:** RNA sequencing data generated in this study were submitted to the Gene Expression Omnibus (GEO) database under accession number GSE59233.

**How to cite this article:** Wolf, C. *et al*. RPA and Rad51 constitute a cell intrinsic mechanism to protect the cytosol from self DNA. *Nat. Commun.* 7:11752 doi: 10.1038/ncomms11752 (2016).

## Supplementary Material

Supplementary Information Supplementary Figures 1-12, Supplementary Tables 1-2, Supplementary Note 1, Supplementary Methods, and Supplementary References

Supplementary Movie 1 Distribution of ssDNA (red) microinjected into one nucleus of a binuclear HEK cell was continuously analyzed by confocal fluorescence microscopy over 30 min. Co-injected dextran (green) marks the microinjected compartment.
