# Supplementary material for: RPA and Rad51 constitute a cell intrinsic mechanism to protect the cytosol from self DNA
Source: Nat Commun. 2016 May 27;7:11752. doi: 10.1038/ncomms11752 (PMC4895045; doi:10.1038/ncomms11752)
Supplement: Supplementary Information — Supplementary Figures 1-12, Supplementary Tables 1-2, Supplementary Note 1, Supplementary Methods, and Supplementary References [file ncomms11752-s1.pdf]

Supplementary Figures

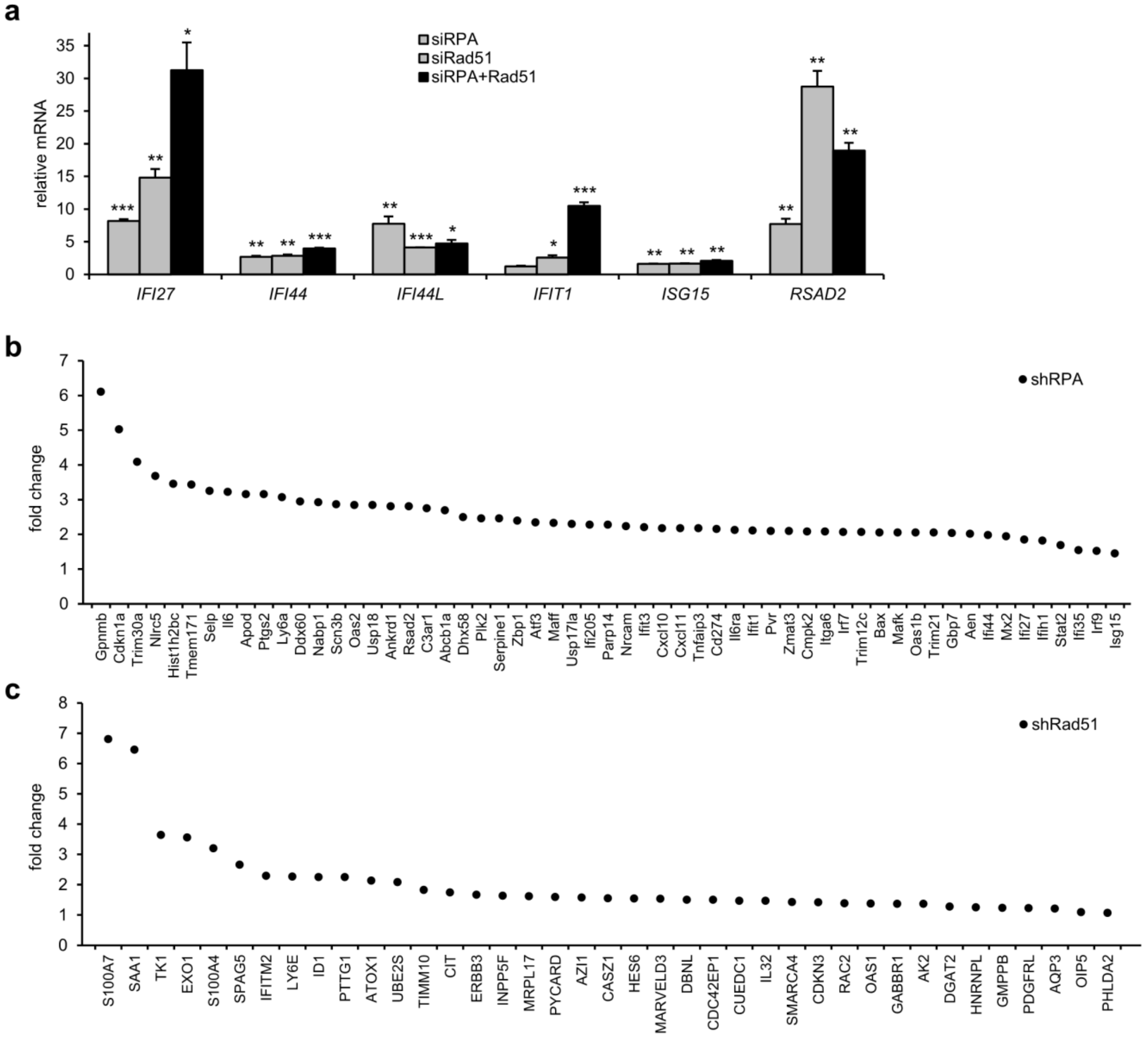

Supplementary Figure 1. Knockdown of RPA and Rad51 leads to ISG induction. (**a**) siRNA-induced knockdown of RPA70 (siRPA) and Rad51 (siRad51) in HeLa cells leads to induction of the interferon-stimulated genes *IFI27*, *IFI44*, *IFI44L*, *IFIT1*, *ISG15* and *RSAD2.* Shown is the fold-change in gene expression relative to a negative control siRNA. Means and s.e.m. of at least two independent experiments run in triplicate. *P < 0.05; **P < 0.01; ***P < 0.001. (**b**, **c**) Transcriptional signatures of ISGs obtained from MEFs with shRNA-induced knockdown of RPA (n = 3) (b) and from human MCF-10A cells with shRNA-induced knockdown of Rad51 (n = 4) (c). Shown are the fold changes in ISG expression for each group relative to controls treated with scrambled shRNA (n = 3 per group) with an adjusted P < 0.05. Transcriptome data were retrieved from the GEO database (accession numbers: GSE38412, GSE54266) and analyzed with GEO2R.

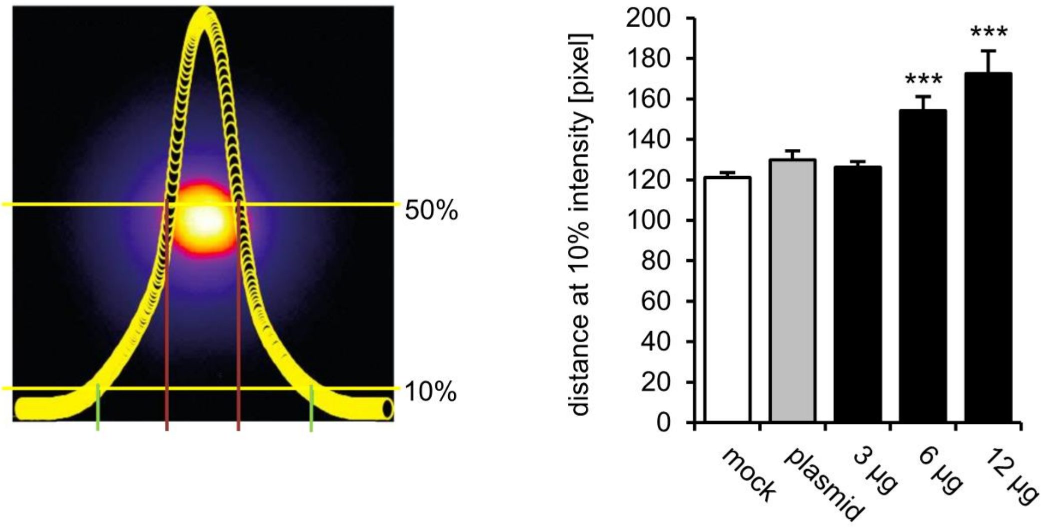

Supplementary Figure 2. Halo assay of fibroblasts transfected with a 45 bp ssDNA oligonucleotide. Schematic of intensity plot used to calculate halo diameters at 10% maximum intensity. Human fibroblasts transfected with increasing amounts of a 45 bp ssDNA oligonucleotide (3, 6, 12 µg) or a 5 kb plasmid (6 µg) show a dose-dependent halo formation. Shown are the means and s.e.m. of two independent experiments run in triplicates. ***$P < 0.001$.

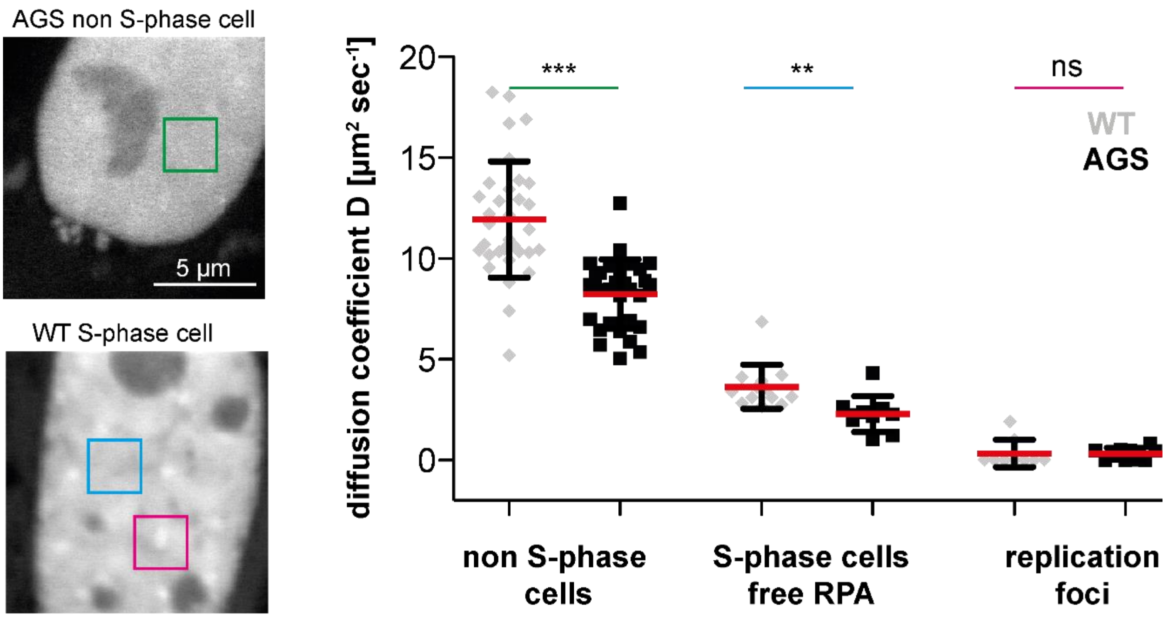

Supplementary Figure 3. Mobility of RPA complexes in living cells measured by raster image correlation spectroscopy (RICS). (**a**) GFP-RPA32 transfected wild type and AGS patient fibroblasts were analyzed by RICS in three distinct conditions. In non S-phase cells (green), in non focal areas in S-phase cells (cyan) and in replication foci (magenta). (**b**) Diffusion coefficients obtained from at least 10 cells per sample. While in wild type cells, RPA34-GFP shows a mean diffusion coefficient of 11.9 + 2.8 $\mu m^2 sec^{-1}$, AGS cells have a reduced diffusion coefficient of 8.2+1.7 $\mu m^2 sec^{-1}$ indicating that RPA is part of a larger complex in AGS cells. Based on the molecular weight, free RPA is expected to have a diffusion coefficient of approximately 30 $\mu m^2 sec^{-1}$ [3]. In S-phase cells, RPA is present in two forms, free RPA and focal RPA engaged in replication. Again the AGS cells exhibit a reduced RPA diffusion (2.2 + 0.9 $\mu m^2 sec^{-1}$), consistent with larger complexes, compared to wild type cells (3.6 + 1.0 $\mu m^2 sec^{-1}$). This fraction is distinct from the RPA fraction engaged in replication foci that exhibits nearly no mobility in both patients and wild type cells (0.3+0.6 vs. 0.3+0.3 $\mu m^2 sec^{-1}$). Red bars represent means, whiskers s.d.. *** $P < 0.001$; ** $P < 0.01$; ns, not significant. ANOVA with Bonferroni´s multiple comparison test.

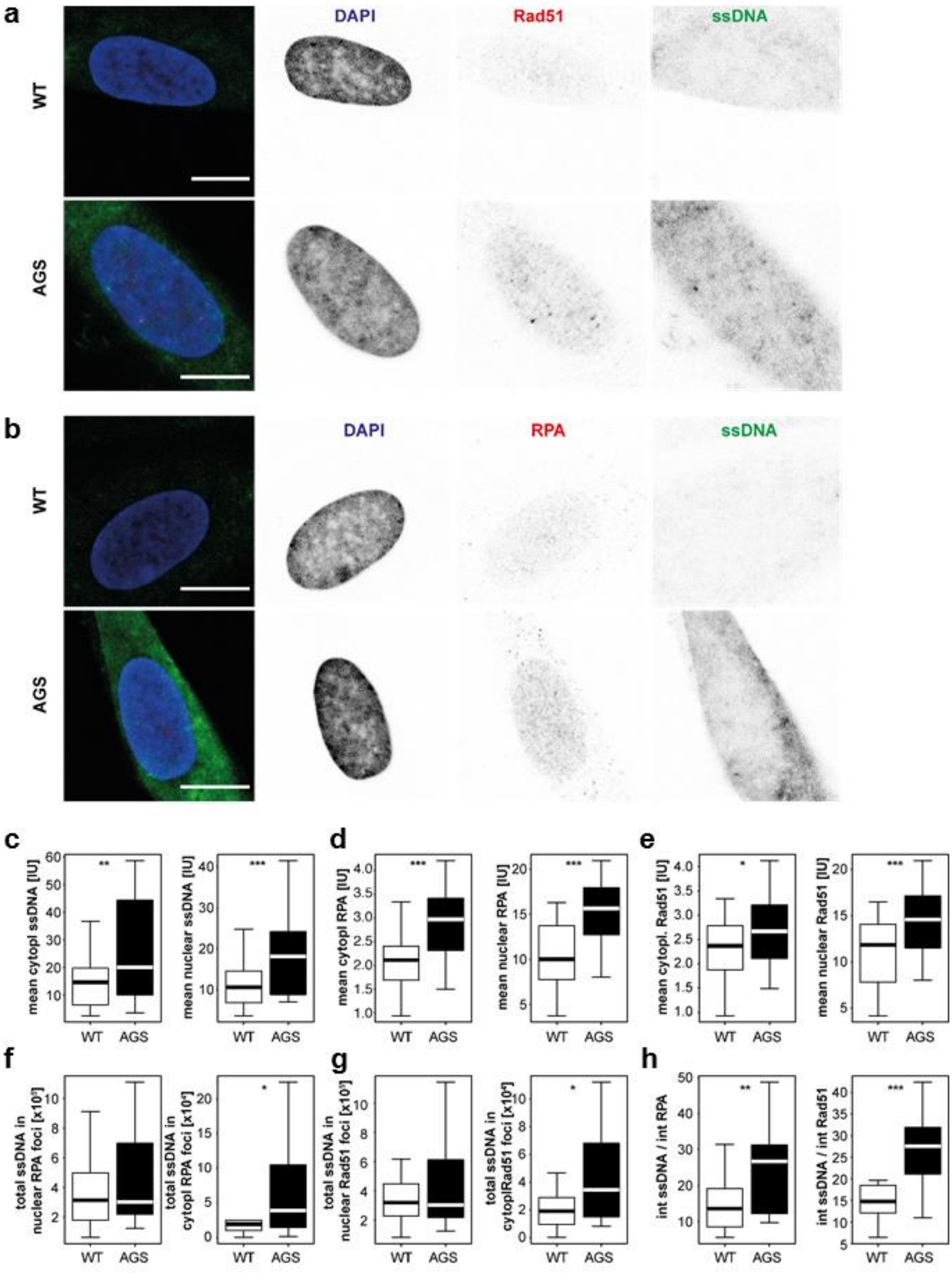
a
DAPI
Rad51
ssDNA
WT
AGS
b
DAPI
RPA
ssDNA
WT
AGS
c
mean cytopl ssDNA [IU]
mean nuclear ssDNA [IU]
d
mean cytopl RPA [IU]
mean nuclear RPA [IU]
e
mean cytopl. Rad51 [IU]
mean nuclear Rad51 [IU]
f
total ssDNA in nuclear RPA foci [x10⁵]
total ssDNA in cytopl RPA foci [x10⁵]
g
total ssDNA in nuclear Rad51 foci [x10⁵]
total ssDNA in cytoplRad51 foci [x10⁴]
h
int ssDNA / int RPA
int ssDNA / int Rad51
WT
AGS

Supplementary Figure 4. Co-staining of ssDNA and RPA or Rad51. (**a**, **b**) Confocal mid nuclear sections of wild type (WT) fibroblasts and TREX1-deficient patient fibroblasts (AGS) co-stained for ssDNA (green) and RPA or Rad51, respectively (red). Nuclei are counterstained with DAPI (blue). AGS cells show enhanced levels of ssDNA compared to WT cells, both in the cytoplasm and the nucleus (**c**). This is accompanied with increased levels of nuclear RPA and Rad51 as well as cytoplasmic RPA and Rad51 (**d**, **e**). Increased co-localization between ssDNA and RPA or Rad51, respectively, is detectable in the cytoplasm, but not in the nucleus (**f**, **g**). The intensity ratios between ssDNA and RPA or Rad51, respectively, are significantly increased in AGS patient cells indicating an exhaustion of the cellular Rad51 and RPA pools (**h**). Boxplots represent the 25th to 75th percentile and whiskers the 5th to 95th percentile. Horizontal lines denote means. * $P < 0.1$, ** $P < 0.01$, *** $P < 0.001$, Student´s t test.

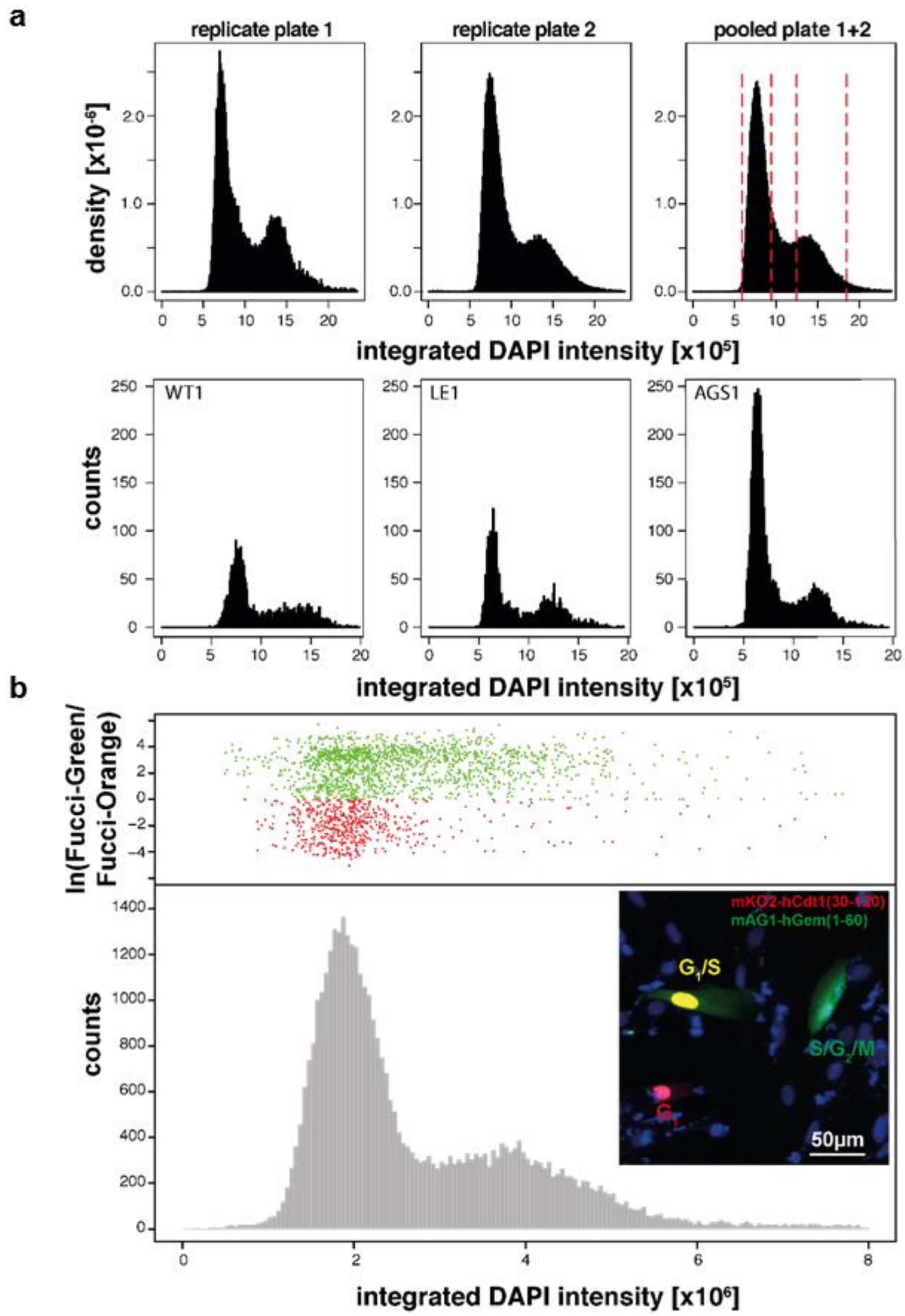

Supplementary Figure 5. Cell cycle analysis by DNA content based on integrated DAPI intensities. (**a**) Comparison of raw data from two different 96-well plates and the pooled dataset (upper panel) as well as from three cell lines (WT1, LE1, AGS1) obtained from single wells (lower panel) demonstrates a stable cell cycle segmentation with clearly identifiable G_1 and G_2 peaks. (**b**) To further validate the DAPI-based cell cycle classification, fibroblasts were transfected with the fluorescent ubiquitination-based cell cycle indicators Kusabira-Orange2-tagged Cdt1 and Azami Green1-tagged Geminin. This results in red nuclei if cells are in G_1 and in green cells if cells are in S, G_2 or M phase. The presence of both colors indicate G_1/S transition. The cell cycle distribution based on integrated DAPI intensities (histogram) corresponds to the distribution of red (G_1) and green (S/G_2/M) fluorescence measured in the same cells.

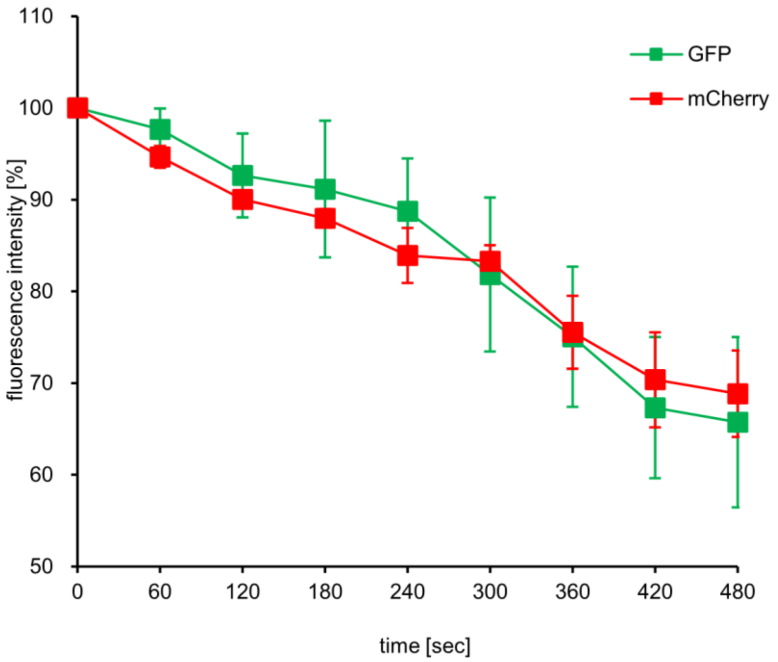

Supplementary Figure 6. Fluorescence intensity decay curves of GFP-TREX1 and TREX1-mCherry. Fluorescence intensities were normalized by setting the highest intensity of each fluorophore to 100%. Shown are mean values and s.d. of triplicate measurements.

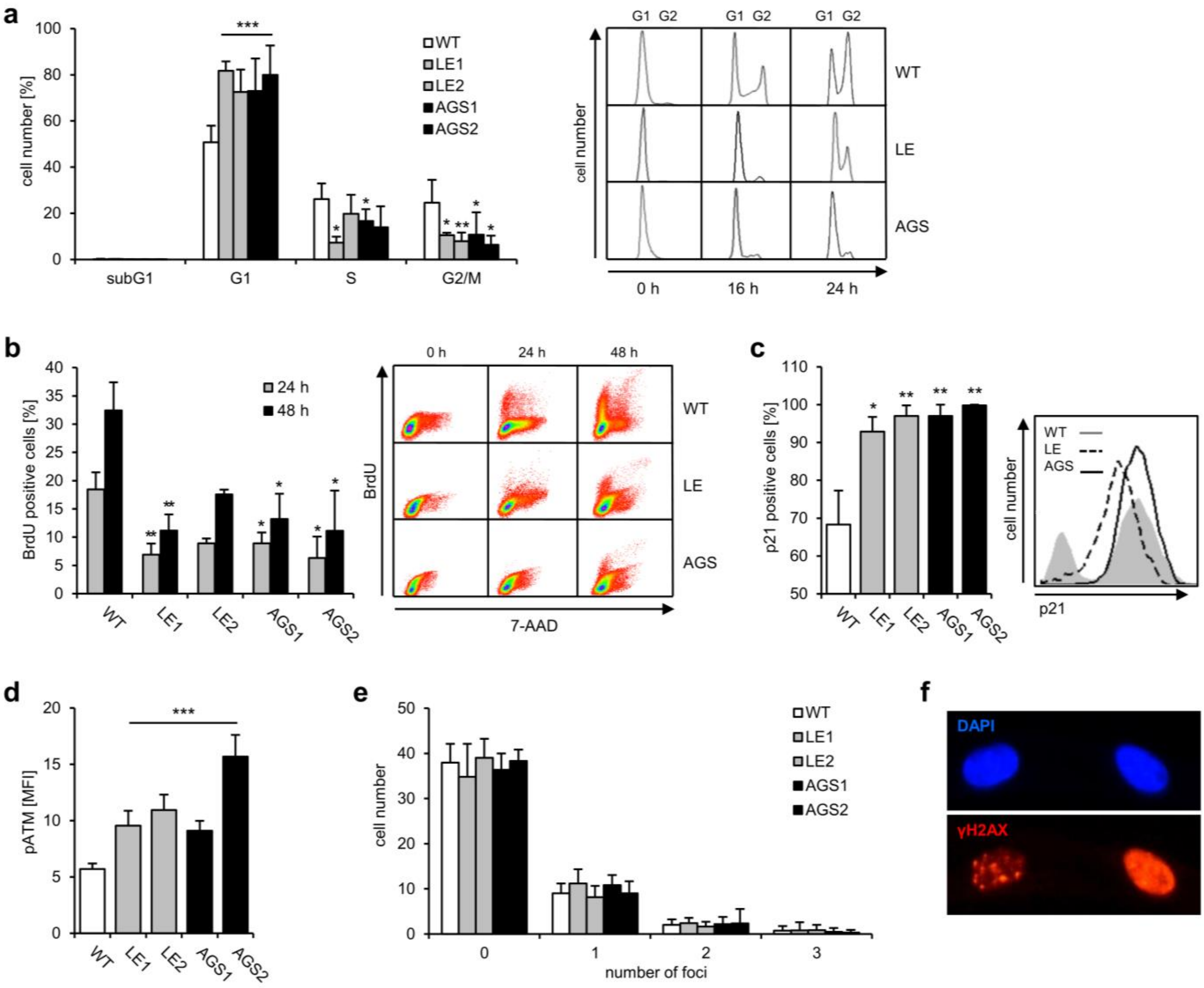

Supplementary Figure 7. Cell cycle delay, DNA damage signaling and checkpoint activation in TREX1-deficient fibroblast. (**a**) Cell cycle analysis of synchronized fibroblasts 16 h after release into growth medium measured by flow cytometry (left). Representative histograms (right). (**b**) Quantification of BrdU-positive fibroblasts showing defective G_1/S transition in TREX1-deficient compared to wild type cells (left). Representative scatter plots of patient (LE, AGS) and wild type (WT) cells 24 and 48 h after BrdU-labeling (right). (**c**, **d**) Flow cytometry of fibroblasts stained for p21 (c) and pATM (d). (**e**) Quantification of DNA double-strand breaks determined by counting of γH2AX and 53BP1-double positive nuclear foci. (**f**) Confocal image of patient fibroblasts showing a nucleus with pan-nuclear staining next to a nucleus with focal staining of γH2AX (red). Nuclei counterstained with DAPI (blue). Shown are the means and s.e.m. of two (c), three (a, b, e) or six (d) independent experiments for each patient (LE1, LE2, AGS1, AGS2) and five wild type controls (WT). **P* < 0.05; ***P* < 0.01; ****P* < 0.01.

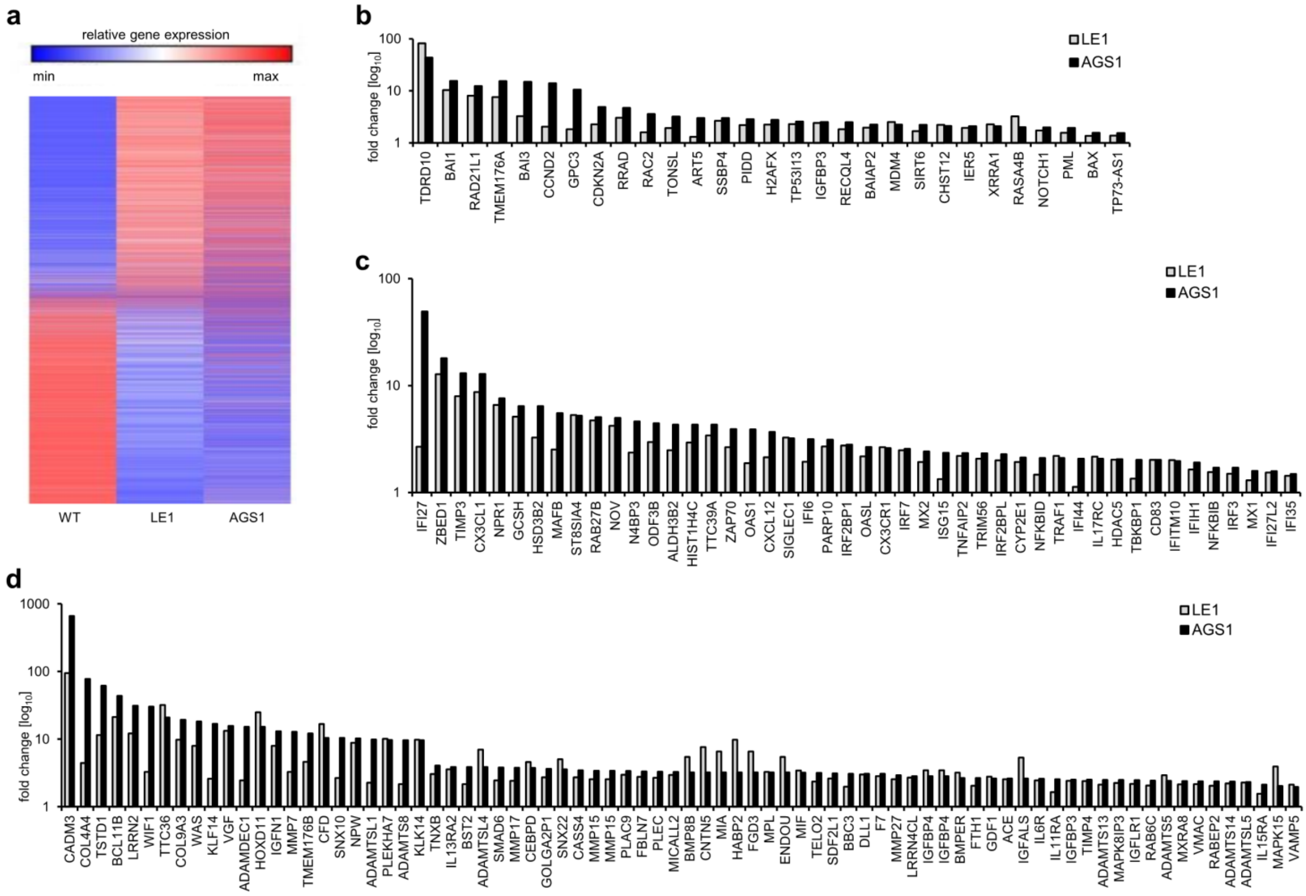

Supplementary Figure 8. Constitutive activation of genes involved in the DNA damage response and of interferon-stimulated genes in TREX1-deficient fibroblasts. (**a**) Transcriptome analysis of patient fibroblasts (LE1, AGS1) and wild type controls (WT, n = 2, mean). The heat map represents over 9000 hierarchically clustered transcripts displaying up-regulation by a factor of at least two. (**b**, **c**, **d**) Fold-changes of the RPKM values of patient fibroblasts (LE1, AGS1) relative to the mean RPKM values of wild type controls (WT, n = 2). Up-regulation of DNA damage response genes (b), interferon-stimulated genes (c) and of genes involved in structural and metabolic changes associated with senescence (d).

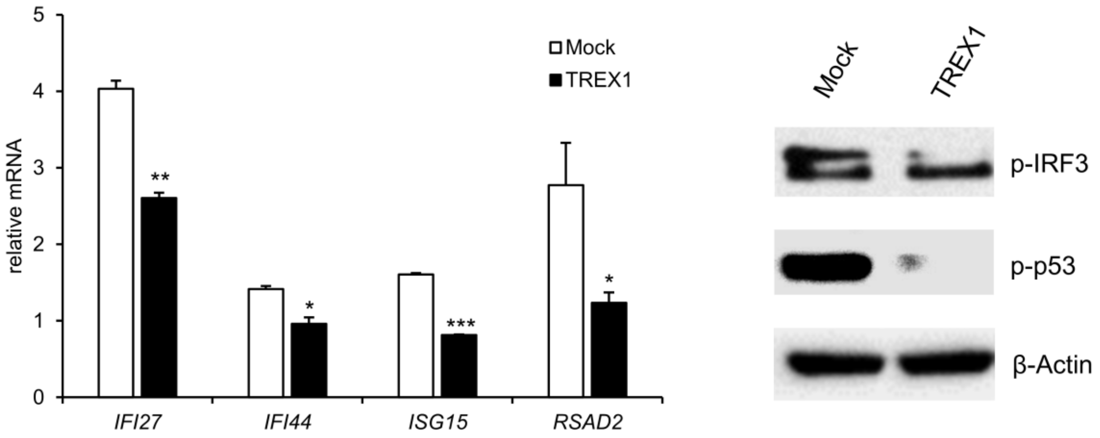

Supplementary Figure 9. Rescue of constitutive activation of type I IFN and DNA damage signaling by overexpression of wild type TREX1 in AGS fibroblasts. TREX1-deficient fibroblasts from an AGS patient transiently transfected with wild type TREX1 (TREX1) exhibit decreased expression of interferon-stimulated genes (left) along with reduced levels of phosphorylated IRF3 (p-IRF3) and p53 (p-p53; right) compared to cells transfected with empty vector (Mock). Quantitative RT-PCR data are presented as means and s.e.m. of one representative experiment run in triplicates out of two independent experiments. *$P < 0.05$; ***$P < 0.01$.

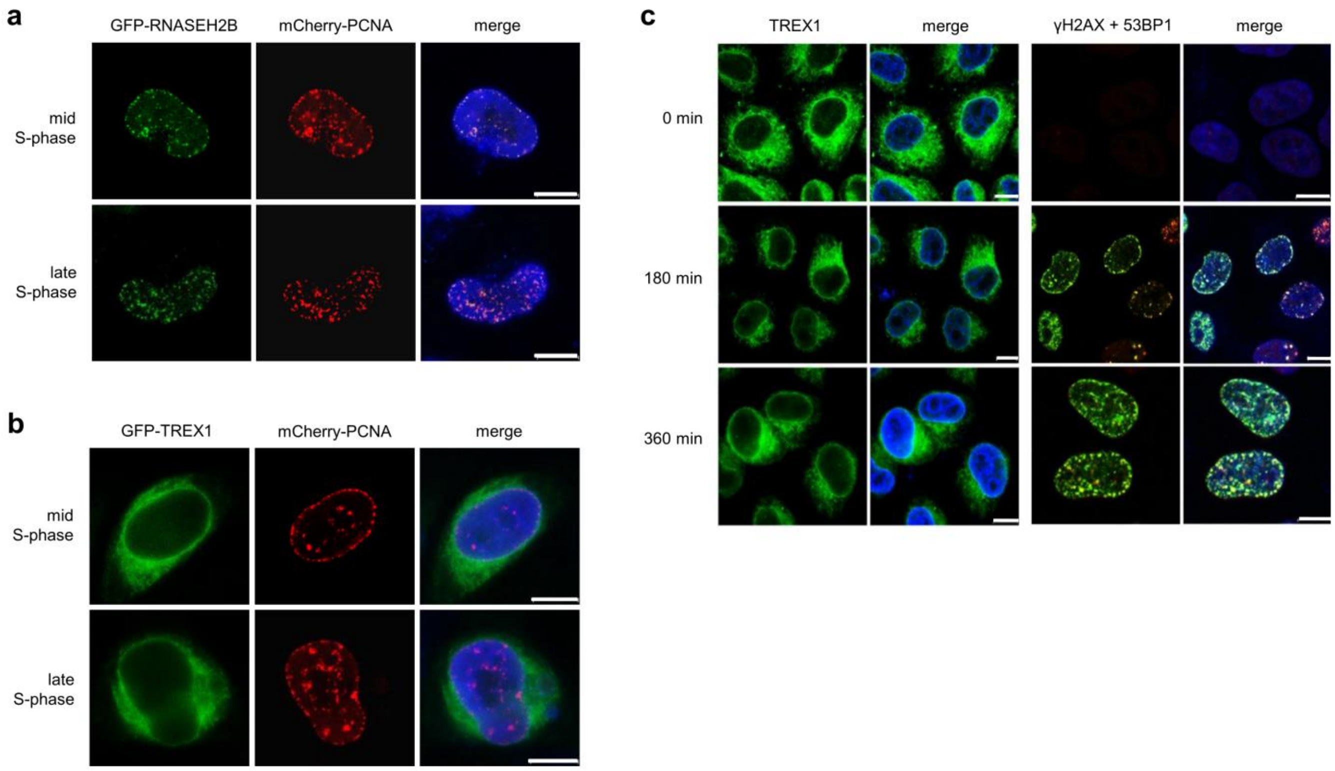

Supplementary Figure 10. TREX1 does not associate with nuclear foci during DNA replication or repair. (**a**, **b**) GFP-RNASEH2B (green, upper panels), but not GFP-TREX1 (green, lower panels), is recruited to replication foci in S-phase nuclei as shown by co-localization with mCherry-PCNA (red) in HeLa cells. Nuclei are stained with DAPI (blue). Scale bars, 10 μM. (**c**) Endogenous TREX1 stained with anti-TREX1 does not associate with DNA damage foci in HeLa cells in response to 6 mM hydroxyurea as shown by immunostaining of γH2AX (green) and 53BP1 (red). Nuclei are stained with DAPI (blue). Scale bars, 10 μM.

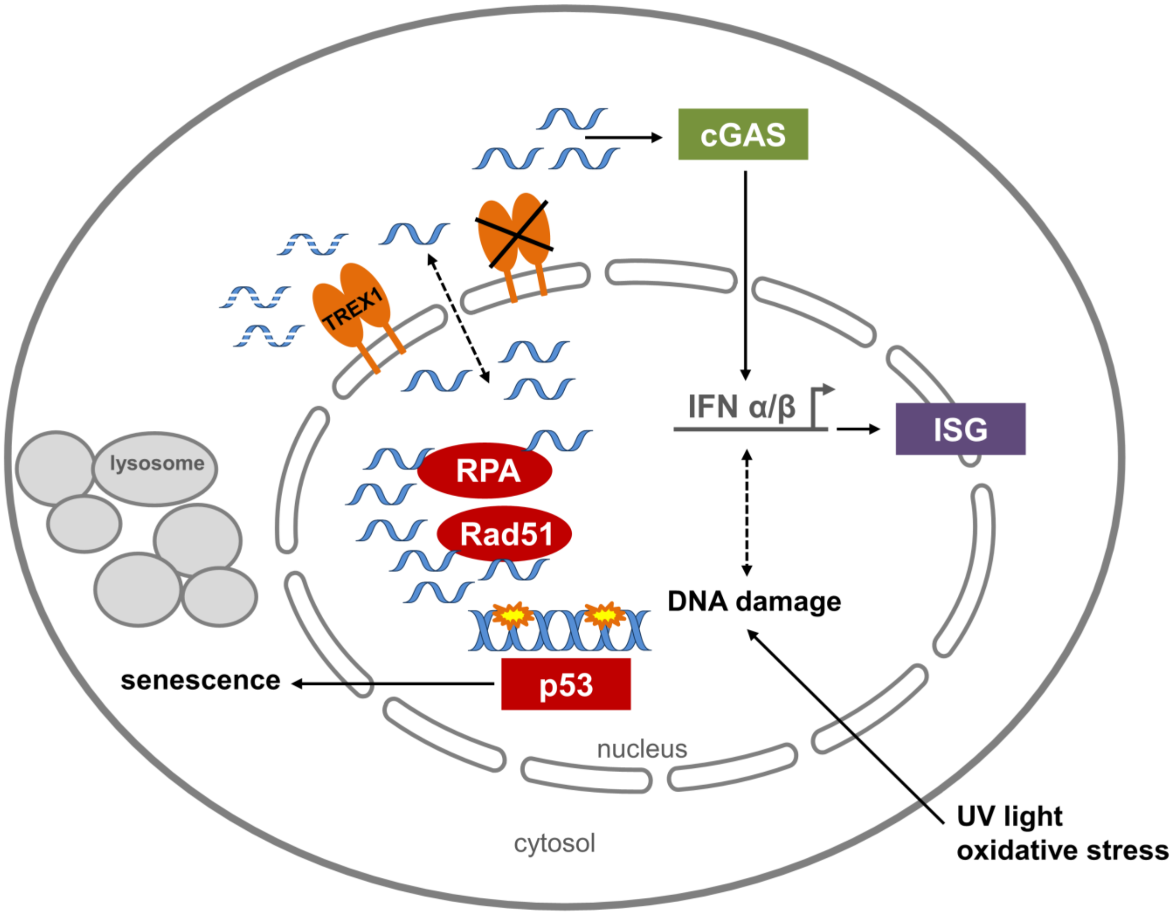

Supplementary Figure 11. Model depicting the role of TREX1 and the ssDNA-binding proteins RPA and Rad51 in the protection of the cytosol from self DNA. Nucleotide excision repair of DNA lesions induced by UV light or oxidative stress results in formation of short ssDNA fragments. The cytosolic tail-anchored nuclease TREX1 degrades nuclear ssDNA that has leaked into the cytosol. In TREX1-deficiency, ssDNA accumulates both in the cytosol and nucleus. In the cytosol, ssDNA is sensed by the pattern-recognition receptor cGAS resulting in type I IFN-dependent innate immune activation. In the nucleus, enhanced ssDNA-binding to RPA and Rad51 causes RPA and Rad51 exhaustion. This leads to unscheduled engagement DNA replication/repair resulting in replication stress and further DNA damage. Chronic low level DNA damage induces a p53-dependent DNA damage response and senescence, which may further contribute to activation of interferon-stimulated genes (ISG).

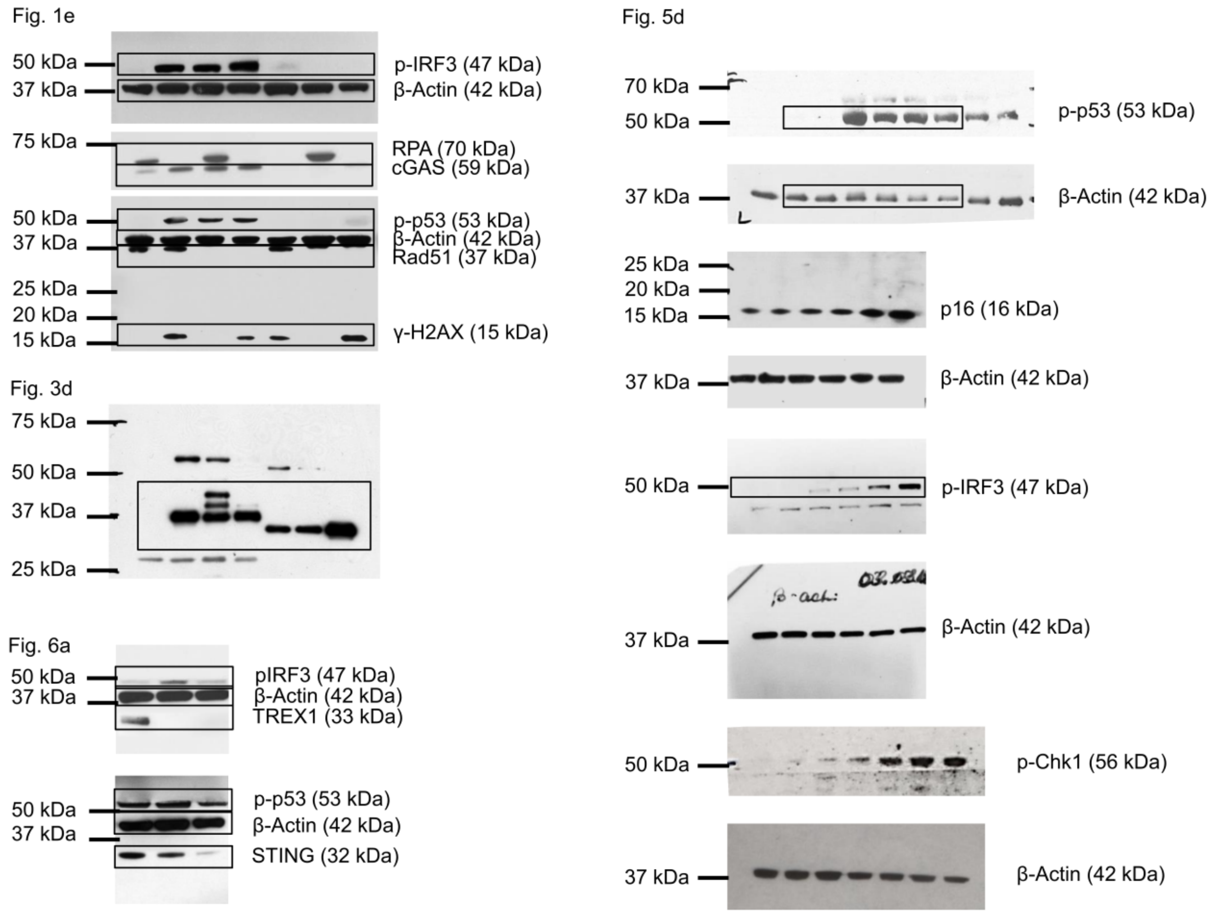

Supplementary Figure 12. Uncropped blots.

Supplementary Tables

Supplementary Table 1. Clinical and laboratory findings in 4 unrelated patients with hereditary breast and ovarian cancer syndrome due to heterozygous germ line mutation in *RAD51C*. The interferon score was determined based on the mean fold change of eight ISGs in each patient in comparison with the median of 10 healthy controls [4].

	**Patient 1**	**Patient 2**	**Patient 3**	**Patient 4**
RAD51C mutation	deletion exon 5-9	deletion exon 5-9	c.224_225insA p.Y75X	c.224_225insA p.Y75X
age	55 years	60 years	76 years	64 years
clinical findings	breast cancer, autoimmune thyroid disease	breast cancer, coronary heart disease	ovarian cancer, diabetes mellitus	ovarian cancer, autoimmune thyroid disease
serological findings	ANA 1:160 homogenous, fine granular [<1:80]		ANA 1:640 homogenous, fine granular [<1:80]<br>C3d-CIC >200 µg/ml [<40]	
interferon score	195.1 [-0.35-0.37]	-29.6 [-0.35-0.37]	274.5 [-0.35-0.37]	-21.4 [-0.35-0.37]

Supplementary Table 2. Oligonucleotides used for quantitative real-time RT-PCR and microinjection. Oligonucleotides were purchased from Eurofins MWG Operon.

Name	Sequence (5' → 3')
IFNB_F	ACCTCCGAAACTGAAGATCTCCTA
IFNB_R	TGCTGGTTGAAGAATGCTTGA
IFNB_P	FAM-CCTGTGCCTCTGGGACTGGACAATTG-TAMRA
IFI27_F	AGCAGTGACCAGTGTGGCCAAAGT
IFI27_R	CTCCAATCACAACTGTAGCAATCC
IFI27_P	FAM-CCTCTGGCTCTGCCGTAGTTTTGCC-TAMRA
IFI44_F	TCTATTCAATACTTCTCCTCTCAGATGATAG
IFI44_R	TGAGCAAAGCCACATGTACCA
IFI44_P	FAM-CCAGCGTTTACCAACTCCCTTCGAATTCTT-TAMRA
GAPDH_F	GAAGGTGAAGGTCGGAGTC
GAPDH_R	GAAGATGGTGATGGGATTTC
GAPDH_P	FAM-CAAGCTTCCCGTTCTCAGCC-TAMRA
ssDNA647N	ATTO647N-ATACGACGGTGACAGTGTTGTCAGACAGGT
ssDNA488	ATTO488-CCTGTCTGACAACACTGTCACCGTCGTATG

Supplementary Notes

Clinical description of patients

Patient 1 (LE1) is a 51 year-old German female with systemic lupus erythematosus and a heterozygous TREX1 mutation (c.634delC; P212fs). She presented with plaques and papules on the back, neck and face since her early forties. Histology of lesional skin revealed perivascular lymphocytic infiltrates and sparse C3 deposits at the dermo-epidermal junction. She complained of photosensitivity, fatigue, recurrent fever and arthralgia of the knee, shoulder, spine and the mandibular joint. At 49 years of age she experienced a transient left sided hemiparesis, which resolved with minimal sequelae. Since that time she suffered from recurrent episodes of migraine. She also developed a depressive psychosis and increasing forgetfulness. Laboratory investigations were remarkable for intermittent lymphopenia (1.25 Gpt/l [1.5-4 Gpt/l]), and elevated γ-glutamate dehydrogenase (1.17 µmol/(s*l) [<0.7]). Antinuclear antibodies (ANA) were present with a maximum titre of 1:640 in a fine-speckled pattern. In addition, she was diagnosed with autoimmune thyroiditis and tested positive for anti-parietal cell antibodies. The patient responded well to hydroxychloroquine and intermittent prednisone in combination with sun protection. Her medical history also included surgical excision of a low grade melanoma on the back, a hysterectomy due to leukoplakia and treatment for invasive ductal carcinoma of the breast. Her family history was remarkable for her mother suffering from stroke and dementia, and her son suffering from photosensitive skin lesions and migraine since early adulthood.

Patient 2 (LE2) is a 45 year-old female from Germany with a familial chilblain lupus due to a heterozygous TREX1 mutation (c.52G>A; D18N). She suffered from cold-induced bluish-red inflammatory skin lesions on her fingers, toes, soles and shins since early childhood. In addition, she complained of arthralgia of knees and shoulders. On histological exam, lesional skin showed periadnexial and perivascular infiltrates. Her laboratory was remarkable for

antinuclear antibodies (1:160) and reduced complement C4 levels (0.15 g/l [0.164-0.392]). Her son and daughter were also affected.

Patient 3 (AGS1) is a 13 year-old boy of Turkish ancestry. His parents are first cousins. He was diagnosed with AGS due to a homozygous V201D (c.602T>A) TREX1 mutation. He was born at term with weight and head circumference within normal range. He presented with dystonia and hypotonia at three months of age. Magnetic resonance imaging (MRI) revealed extensive calcification of basal ganglia, myelin defects and atrophy. A lumbar puncture demonstrated lymphocytosis and raised IFN-α. He developed microcephaly, spastic tetraparesis and severe global developmental delay. He also suffered from chilblain lesions on the toes. He was diagnosed with type 1 diabetes mellitus at 7 years. At 10 years of age, he tested positive for ANA (1:160), anti-ENA (extractable nuclear antigens), cardiolipin-IgG 41.51 U/ml [<10] and anti-dsDNA 77.62 IU/ml [<30].

Patient 4 (AGS2) is a 19 year-old German male patient who carries the common R114H (c.341G>A) TREX1 mutation in the homozygous state. He was born at 36 weeks of gestation; his weight and head circumference were within normal limits for gestational age. He presented with hypotonia, seizures and dystonia at 4 months of age. He was found to have elevated IFN-α in cerebrospinal fluid. His MRI showed a leukencephalopathy with enlarged ventricles. He developed microcephaly and severe developmental delay. At the age of 17 years serologic testing revealed an ANA titre of 1:1280. He also tested positive for: anti-nucleosome 138.36 U/ml [<30], cardiolipin-IgG 15.5 U/ml [<10] and C3d circulating immune complexes 49.24 μg/ml [<40].

Supplementary Methods

Immunolabeling for correlative light and electron microscopy. GFP-TREX1-transfected cells were fixed with 4% paraformaldehyde (PFA) in 0.1 M phosphate buffer (PB, pH 7.4) and processed for Tokuyasu cryo-sectioning using a Leica UC6 ultramicrotome equipped with a FC6 cryo-chamber. Dry-frozen ultrathin sections were picked up in methyl cellulose sucrose (1 part 2% methyl cellulose (Sigma) + 1 part 2.3 M sucrose) and transferred onto an electron microscopic support grid for subsequent immunogold labeling. Grids were placed upside down on drops of PBS, washed 5 times with 0.1% glycin/PBS, blocked with 1% BSA/PBS and incubated with anti-GFP-antibody (TP-401 from Torrey Pines; 1: 100) for 1 h. After washes in PBS, the sections were incubated with Protein A conjugated to 10 nm gold for 1 h, washed again in PBS and postfixed in 1% glutaraldehyde. Grids were washed several times in water, stained with neutral uranyl oxalate (2% UA in 0.15 M oxalic acid, pH7.0) for 5 min, washed shortly in water and incubated in methyl cellulose uranyl acetate (9 parts 2% MC + 1 part 4% UA, pH 4) on ice for 5 min. Finally, grids were looped out, the MC/UA film was reduced to an even thin film and air dried. The sections were analyzed on a Philips Morgagni 268 (FEI) at 80 kV and images were taken with the MegaView III digital camera (Olympus).

Western blot analysis, β-galactosidase staining, IFN reporter assay. Cells were lysed in RIPA buffer (50 mM Tris-HCl, pH 7.4, 150 mM NaCl, 1 mM EDTA, 1% Triton X-100, 1 mM sodium orthovanadate, 20 mM sodium fluoride) supplemented with 1x Complete Protease Inhibitor Cocktail and 1x PhosSTOP phosphatase inhibitors (Roche). Protein concentration was determined using a BCA Kit (Thermo Scientific). Lysates were subjected to SDS-PAGE and blotted onto a nitrocellulose membrane (BA83, Sigma-Aldrich). Membranes were blocked in 5% dry milk and probed using the following antibodies: anti-p53 (Ser15, sc-101762, Santa Cruz; 1:200), anti-p16 (sc-468, Santa Cruz; 1:1,000), anti-Chk1 (Ser345, 2348, Cell Signaling; 1:1,000), anti-pIRF3 (Ser386, ab76493, abcam, 1:500), anti-Rad51 (PC130, Merck; 1:500), anti-RPA (Cell Signaling, 1:1,000), anti-cGAS (Cell Signaling, 1:1,000) and anti-β-actin (clone AC-74, Sigma Aldrich; 1:10,000). Immunoreactive signals were detected by

chemiluminescence (Lumi-Light PLUS, Roche). Fibroblasts were stained using the Senescence β-Galactosidase Staining Kit (Cell Signaling) and analyzed by light microscopy. For quantification, cells of 8 randomly chosen fields were counted. IFN-β was measured by using HEK-Blue IFN-α/β cells (InvivoGen).

Cell cycle analysis by quantitative DNA imaging. Cell cycle analysis was performed by DNA content analysis based on the integrated DAPI intensity per nucleus [7]. At least 100 fields of view were imaged per sample point using the Operetta high content imager (Perkin Elmer) equipped with a bandpass filter for DAPI (excitation: 360-400 nm; emission: 410-480 nm) using constant exposure times of 50 ms. Nuclear segmentation was performed by the Harmony software (Perkin Elmer) using method M and a guide size of 15 µm diameter per nucleus. A split factor of 0.1 and local threshold were applied. Then the total nuclear DAPI intensity was measured. For fluorescent ubiquitination-based cell cycle indicator (Fucci) analysis [8], fibroblasts were transiently transfected with the two cell cycle reporter plasmids Fucci-G_1 Orange (monomeric Kusabira-Orange2-tagged Cdt1; Amalgaam Co.) and Fucci-S/G_2/M (monomeric Azami Green1-tagged geminin; Amalgaam Co.). While Fucci-G_1 Orange contains aa 30-120 of human Cdt1 and is only expressed in G_1, Fucci-S/G_2/M Green contains aa 1-60 of human Geminin and stains both nucleus and cytoplasm in green after G1/S transition. Again the Operetta high content imager was used to record the images of DAPI as well as of monomeric Azami-Green1 (excitation: 460-490 nm; emission: 500-550 nm; exposure 300 ms) and monomeric Kusabira-Orange2 (excitation: 560-580 nm; emission: 590-640 nm; exposure 300 ms). Nuclear segmentation was done as described above and nuclear intensities in the red and green channel were measured accordingly. Cells expressing at least one of the two constructs were selected and plotted against the integrated DAPI intensity within the nucleus.

Immunohistochemistry. Fibroblasts grown on coverslips were fixed with 4% paraformaldehyde for 10 min and permeabilized for 5 min using 0.2% Triton X-100. To analyze

the detergent-extractable fraction of RPA, cells were first permeabilized for 5 min with 0.2% Triton X-100 followed by fixation with 4% paraformaldehyde for 10 min. After blocking with 1% BSA in PBS, slides were incubated with the following primary antibodies: anti-Rad51 (PC130, Merck; 1:500), anti-RPA (Cell Signaling, 1:1000). Slides were washed with 0.2% Tween in PBS and then incubated with the respective Alexa 488-conjugated secondary antibody (Invitrogen). Cells were mounted with VectaShield containing DAPI (Vector Labs). For high content automated imaging (Operetta, Perkin Elmer), a minimum of 100 fields of view were imaged per experimental sample using a 40x 0.95 NA air objective and analyzed with the Harmony software (Perkin Elmer). The following analysis sequence was applied: identify nuclei based on DAPI staining (method B), identify spots (method A) with relative spot intensity >0.04 and splitting coefficient =1. For each spot, contrast and mean intensity were calculated and spots with a intensity spot/spot area ratio >0.0015 selected to exclude nucleolar and weak background staining. In addition, the total nuclear intensity of RPA and Rad51 staining was measured. For staining of ssDNA, cells were first incubated with 0.1 mg/ml RNase A (Biozyme) in RNase A Buffer at 37°C for 30 min followed by incubation with or without 20 U/ml S1 nuclease in S1 nuclease-buffer (Applied Biosystems) at 37°C for 30 min. After blocking with 1% BSA in PBS, cells were stained with anti-ssDNA antibody (ABIN180204; 1:200). The signal was amplified by the secondary antibody using the Tyramide Signal Amplification Kit (Molecular Probes) and the mean fluorescence intensity from at least 65 cells per slide was determined using Image J. For subcellular analysis of TREX1, cells were treated with 6 mM hydroxyurea (Sigma) for 180 and 360 min, respectively, and then stained with anti-TREX1 (122115, Cell Signaling, 1:100) followed by the secondary antibody using the Tyramide Signal Amplification Kit (Molecular Probes). For staining of DNA double-strand breaks, fibroblasts or HeLa cells stably expressing mCherry-PCNA [1] were transfected with 200 ng of GFP-TREX1 or GFP-RNase H2B, respectively, and then probed with anti-γH2AX (Ser139, clone 2F3, Novus Biologicals; 1:500) anti-53BP1 (NB100-904, Novus Biologicals; 1:2,000) followed by incubation with Alexa Fluor 546-conjugated goat-anti-mouse-IgG and Alexa Fluor 488-conjugated goat-anti-rabbit-IgG (Molecular Probes). Cells were mounted in VectaShield

Mounting Medium with DAPI (Vector Labs) and analyzed with a Zeiss Axioimager A1 microscope. For quantification, double stained foci in 50 randomly selected cells were counted per slide. For detection of CPDs, cells were fixed with methanol (-20°C) and acetone. DNA was denatured in 0.1 N NaOH. Cells were then dehydrated with increasing concentrations of ethanol (70-100%) and incubated with anti-CPD (clone KTM53, Kamiya Biomedical Company; 1:1,000) followed by staining with Alexa Fluor 488-conjugated goat-anti-mouse-IgG (Molecular Probes). The mean fluorescence intensity from 50 cells per slide was determined using Image J. For co-staining of RPA / Rad51 and ssDNA cells were imaged using a confocal laser scanning microscope (Leica SP5 II) equipped with HyD detectors operated in single photon counting mode. A confocal mid nuclear section was imaged using a CX PL APO 63x and images were quantified using Volocity (Perkin Elmer), a minimum of 10 cells were analyzed in two wild type cell lines and two AGS patient cell lines.

Raster image correlation spectroscopy. 2 x 10^4 cells per well were seeded in fibronectin-coated chambered glass slides (Nunc Lab-Tek), transfected with 10 ng of GFP-RPA32 [10] using FuGENE HD (Promega) and subjected to live cell imaging after 48 h. Raster image correlation spectroscopy (RICS) [2] was performed on an Zeiss confocal laser scanning microscope LSM 780 Axio Observer of the Light Microscopy Facility, a core facility of BIOTEC/CRTD at Technische Universität Dresden. Data were acquired and analyzed with the Zen 2.1 software (Zeiss). In detail, 64 x 64 pixel areas within the nucleus were analyzed (2-5 areas per nucleus) and spatial autocorrelation was calculated after detrending (immobile structures and stack) using the 3Dcoeff default formula for RICS. Each cell was visually inspected for focus drift and goodness of fit of the autocorrelation function. Time series were acquired with a pixel size of 51 nm and a pixel dwell time of 6.3 µs using the 488 nm line of an Argon ion laser. A minimum of 10 cells was analyzed from each of two wild type and two AGS fibroblast cell lines.

Sytox Orange assay. HeLa-cells transfected with YFP-TREX1 were fixed in 4% paraformaldehyde and permeabilized with PBS containing 0.5% Triton-X 100 and 100 mM

glycine to block aldehyde groups. Digestion of nucleic acids was performed with 20 U/ml DNase I in DNase I-buffer (Applied Biosystems) at 37°C for 30 min. Cells were then stained with 5 µM Sytox Orange (Molecular Probes) for 30 min, washed and mounted with VectaShield containing DAPI (Vector Labs). Fluorescence life time imaging analysis was carried out in the cytoplasm as previously described [11] using a Zeiss Axiovert S100TV with a BL HC513/17 emission band-pass filter (Semrock). At least 20 cells were measured per experiment.

Microinjection, fluorescence cross-correlation spectroscopy. Cells were grown in MaTek chambers. Immediately before microinjection, the growth medium was replaced by air-buffer (150 mM NaCl, 20 mM HEPES pH 7.4, 15 mM Glucose, 150 µg/ml BSA, 20 mM trehalose, 5.4 mM KCl, 0.85 mM $MgSO_4$, 0.6 mM $CaCl_2$). The micropipette (Femtotip 2, Eppendorf) was loaded with 2.5 µl injection solution (110 mM K-gluconate, 18 mM NaCl, 10 mM Hepes pH 7.4, 0.6 mM MgSO4) containing either 5 µM ssDNA647N and 4 µM 150 kDa FITC-dextran or 5 µM dsDNA488_647N and 5 µM 70 kDa Rhodamine B-dextran, respectively. Oligonucleotides were from IBA Life Sciences. Microinjection was carried out on an LSM780-Confocor3 microscope equipped with a microinjector consisting of a Femtojet and InjectMan NI2 (Eppendorf) mounted directly inside an incubation chamber at 37°C. Working pressure for injection was between 20-40 hPa for 0.1s and a holding pressure of 15 hPa. Fluorescence cross-correlation spectroscopy (FCCS) was used to assess exonuclease activity within the cytosol of living cells. FCCS is a highly sensitive biophysical technique for the study of the dynamics and interactions of two differently labeled molecule species in living cells. Two superimposed laser lines of different wavelength are focused by a single microscope objective in order to generate overlapping focal volume elements. In this way, two spectrally distinct dyes can be simultaneously excited and their fluorescence emission detected via the same objective in two separate channels. In fluid environments, diffusion of fluorescent molecules through the focal spot induces fluorescence fluctuation. Both fluctuating fluorescence signals are subjected to auto-correlation analysis. Additionally, the calculated cross-correlation function is obtained by correlating intensity fluctuations of both channels with each other. Only

when a dual-labeled molecule translocates through the detection volume it contributes to the cross-correlation curve. Hence, the amplitude of the cross-correlation curve is directly proportional to the amount of dual-labeled species and allows a quantitative analysis of interactions. Given that TREX1 also degrades dsDNA with 3´overhangs, we used a dual-labeled dsDNA oligonucleotide with 3´overhangs as substrate and measured exonucleolytic activity as loss of cross-correlation over time. FCCS was carried out directly after microinjection on the same microscope. Images were taken with low laser powers with spectral GaAsP-detector in photon counting mode. The 488 nm Ar-laser-line was used to excite ATTO488 and the 633 nm line was used to excite ATTO647N. Both laser lines were attenuated by an acousto-optical tunable filter to an intensity in the focal plane of 5.3 and 1.4 kW/cm², respectively, in order to minimize photobleaching and cellular damage. Both excitation laser lines were directed by a 488/633 dichroic mirror (MBS) onto the back aperture of a Zeiss C Apochromat 40x, N.A.=1.2, water immersion objective. The fluorescence light was collected by the same objective, separated from the excitation light by the MBS, passing a confocal pinhole (35 µm in diameter) and split into two spectral channels by a second dichroic (NFT, LP635). After removing residual laser light by a 505-610 nm bandpass and 655 nm longpass emission filter, respectively, the fluorescence light was recorded by avalanche photodiode detectors (APDs). The setup was adjusted using a dye mixture of Alexa488 and Alexa633. Before each experiment the amount of cross-correlation for the injected dsDNA (100 nM in injection buffer) was measured and used for normalization. The overall cross-correlation performance was determined with a 198 bp long ATTO488/ATTO647N double-labeled DNA yielding cross-correlation amplitudes of 75% (±5%). The discrepancy to 100% is due to imperfect overlap of the detection volumes and/or imperfect labeling of the DNA. FCCS was measured in the cytoplasm. At least 10 cells were measured per experiment in at least two independent experiments. Measurements were started as fast as possible after microinjection (approx. 15 min after injection start). For each measurement, 24 runs, each 10 sec long, were collected. The fluorescence signals of each run were software-correlated following the definition of auto- and cross-correlation as previously described [6].

Comet assay, halo assay. Alkaline single cell gel electrophoresis was performed as described [5]. For halo assays [9], frosted microscope slides were pre-coated with melted 0.5% agarose (type II, Sigma) in PBS followed by 1% agarose (type II, Sigma). 1.5 x 10^4 cells synchronized by 24 h serum starvation were suspended in 150 µl 3% low-melting point agarose (type VII, Sigma) melted in PBS at 50°C and dispersed on pre-warmed pre-coated slides. Fibroblasts were transfected with the indicated amount of a 45 bp oligonucleotide or plasmid DNA using polyethylenimine prior to synchronization. After gelling, slides were incubated in alkaline lysis buffer (2.5 M NaCl, 0.1 M EDTA, 10 mM Tris, 1% Triton X-100, pH 10) at 4°C for 18 h. Slides were then washed 10 times for 10 min in PBS. After neutralization in 0.5M Tris-HCl for 30 min, 200 µl Antifade with Sybr Gold (20 mM DABCO, 0.1 µl/ml Sybr Gold, 20 mM Tris-HCl, 80% glycerol) was added to each slide and covered with a cover slip. Microscopy was carried out at 10x magnification. Images were captured using constant exposure times and a minimum of 50 cells per slide was analyzed using Image J. Single nuclei were cropped and adjacent background was subtracted. A vertical and horizontal intensity profile was measured. Based on the intensity profile the distance between the 10% maximum intensities of the left and right flanks (upper and lower, respectively) was calculated for each nucleus. For cumulative curves the intensity profiles were centered to the maximum position and normalized to the maximum intensity value. Then mean profiles were calculated for each population.

RNA sequencing. RNA sequencing was performed as previously described [5]. RNA sequencing data were submitted to the Gene Expression Omnibus (GEO) under accession number GSE59233. Transcriptome analysis of shRNA-induced knockout of RPA in MEFs (GSE38412) and of Rad51 in MCF-10A mammary epithelial cells (GSE54266), respectively, was carried out using published datasets retrieved from the GEO database (www.ncbi.nlm.nih.gov/geo). Differential gene expression was analyzed using GEO2R by comparing gene expression profiles of each group (n = 3 for RPA; n = 4 for Rad51) with those

of the respective control groups treated with scrambled shRNA (n = 3 per group). Fold changes in ISGs expression with an adjusted $P < 0.05$ were considered significant.

Supplementary References

1. Chagin, V. O., Casas-Delucchi, C. S., Reinhart, M., Schermelleh, L., Markaki, Y. Maiser, A., Bolius, J.J., Bensimon, A., Fillies, M., Domaing, P., Rozanov, Y.M., Leonhardt, H. & Cardoso, M.C. 4D Visualization of replication foci in mammalian cells corresponding to individual replicons. *Nat. Commun.* **7,** doi:10.1038/ncomms11231 (2016).
2. Digman, M. A., Brown, C. M., Sengupta, P., Wiseman, P. W., Horwitz, A. R. & Gratton, E. Measuring fast dynamics in solutions and cells with a laser scanning microscope. *Biophys. J.* **89,** 1317-1327 (2005).
3. Dross, N., Spriet, C., Zwerger, M., Muller, G., Waldeck, W. & Langowski, J. Mapping eGFP oligomer mobility in living cell nuclei. *PLoS. One.* **4,** e5041 (2009).
4. Feng, X., Wu, H., Grossman, J. M., Hanvivadhanakul, P., FitzGerald, J. D., Park, G. S. *et al.* Association of increased interferon-inducible gene expression with disease activity and lupus nephritis in patients with systemic lupus erythematosus. *Arthritis Rheum.* **54,** 2951-2962 (2006).
5. Kretschmer, S., Wolf, C., Konig, N., Staroske, W., Guck, J., Hausler, M. *et al.* SAMHD1 prevents autoimmunity by maintaining genome stability. *Ann. Rheum. Dis.* **74,** e17 (2015).
6. Ohrt, T., Staroske, W., Mutze, J., Crell, K., Landthaler, M. & Schwille, P. Fluorescence cross-correlation spectroscopy reveals mechanistic insights into the effect of 2'-O-methyl modified siRNAs in living cells. *Biophys. J.* **100,** 2981-2990 (2011).
7. Roukos, V., Pegoraro, G., Voss, T. C. & Misteli, T. Cell cycle staging of individual cells by fluorescence microscopy. *Nat. Protoc.* **10,** 334-348 (2015).
8. Sakaue-Sawano, A., Kurokawa, H., Morimura, T., Hanyu, A., Hama, H., Osawa, H. *et al.* Visualizing spatiotemporal dynamics of multicellular cell-cycle progression. *Cell* **132,** 487-498 (2008).
9. Sestili, P. The fast-halo assay for the assessment of DNA damage at the single-cell level. *Methods Mol. Biol.* **521,** 517-533 (2009).

10. Sporbert, A., Gahl, A., Ankerhold, R., Leonhardt, H. & Cardoso, M. C. DNA polymerase clamp shows little turnover at established replication sites but sequential de novo assembly at adjacent origin clusters. *Mol. Cell* **10,** 1355-1365 (2002).

11. Tungler, V., Staroske, W., Kind, B., Dobrick, M., Kretschmer, S., Schmidt, F. *et al.* Single-stranded nucleic acids promote SAMHD1 complex formation. *J. Mol. Med. (Berl)* **91,** 759-770 (2013).
